# Experimental and numerical study of tubular steel columns with/without demountable bolted shear connectors embedded in the concrete

**DOI:** 10.1038/s41598-025-94227-3

**Published:** 2025-04-26

**Authors:** Sabry Fayed, Moataz Badawi, Mohamed Ghalla, Ehab A. Mlybari, Yahia Iskander, Saad A. Yehia

**Affiliations:** 1 Department of Civil Engineering, Faculty of Engineering, Kafrelsheikh University, Kafrelsheikh, Egypt; 2 Department of Civil Engineering, College of Engineering and Architecture, Umm Al-Qura University, Makkah, Saudi Arabia; 3 Civil Engineering Department, Higher Institute of Engineering and Technology, Kafrelsheikh, Egypt

**Keywords:** Normal concrete, Demountable bolted shear connectors, Steel tubular columns, Push-out test, Shear bearing capacity, 3D model, Engineering, Materials science

## Abstract

Three push-out specimens were experimentally tested to investigate the behavior of tubular steel columns (TSC) with and without bolted shear connectors embedded in normal concrete (NC). Each specimen consisted of a tubular steel column (TSC) encased in a 250 × 250 × 200 mm concrete cube The embedment/the prominent height of TSC was 100 mm. Foam was used underneath the TSC to form free space. The study considered variables such as the presence of demountable shear studs and reinforcement. The failure modes, load-slip response, peak load/slip, and shear stiffness of the specimens were analyzed. Furthermore, a finite element model (FEM) was developed using ABAQUS software to simulate the behavior of the tested specimens and validated against the experimental results. The FEM was also employed to conduct further parametric investigations. The results indicate that demountable shear studs significantly improve shear capacity, with specimens exhibiting a 217% higher peak load than those without studs. Reinforcing the concrete block had a negligible effect on peak load but increased peak slip by 37.7% and shear stiffness by 18.7% compared to the unreinforced specimen. Furthermore, increasing the TSC thickness significantly enhances peak load, with a 154.31% increase observed as the thickness increases from one-third of the bolt diameter to the full bolt diameter. Additionally, using TSC thicknesses greater than half the bolt diameter helps prevent bearing failure. Increasing the concrete compressive strength from 25 to 50 MPa leads to a 24.6% increase in peak load, while slip capacity decreases by 19.77%. For applications requiring high ductility, excessively high-strength concrete should be avoided, as it reduces slip capacity. The results also demonstrate that the bolt diameter should not exceed twice the TSC web thickness to prevent bearing failure.

## Introduction

Recently, the adoption of composite structures has increased globally, leveraging the strengths of both steel and concrete, with significant applications in bridges^1,2^, tall buildings, and steel structures^3–5^. Concrete-steel composite structures provide key benefits, such as higher capacity, lower deformation, and the option for more compact sections^6–8^. These composite structures can be constructed with shear connectors, either welded studs or bolted, which enhance the distribution of internal stresses^9^. Welded studs are widely used to create composite action, whereas bolted connectors are ideal for smaller composite sections due to their simplicity, ease of installation, and strong resistance^10^. Furthermore, metal sections are reusable; however, if welded to a concrete slab, they cannot be reused^11^. A demountable bolted connection facilitates composite behavior and enables the reuse of both elements.

However, despite these advantages, existing design codes impose several limitations on the use of bolted steel–concrete connections. ACI 318^12^ lacks explicit provisions for bolted shear connections and fatigue behavior, focusing more on embedded anchors. Eurocode 4^13^ assumes stud connectors as the primary shear transfer mechanism, oversimplifies slip effects, and provides limited experimental validation. AISC 360^14^ primarily addresses steel connections, with minimal guidance on concrete contributions and bolt slippage. AS/NZS 2327^15^ restricts bolt types and assumes rigid load transfer, leading to conservative safety factors. Common issues across codes include inadequate provisions for high-strength concrete, limited dynamic load considerations, and oversimplified shear-moment interactions.

In response to these limitations, researchers have explored the mechanical performance of high-strength bolted joints in steel–concrete composite beams, investigating their strength, slip behavior, and long-term performance**.** Dai et al.^16^ conducted a series of push tests on bolted connectors machined from studs, demonstrating a high slip capacity and shear resistance reaching 84% of that of welded studs at a slip displacement of 6 mm. Pavlović et al.^17^ found that bolted shear connectors achieved 95% of the shear strength of welded headed studs, but with 50% less stiffness, making bolted connectors a viable yet less rigid alternative to welded studs. Moynihan and Allwood^11^ performed three tests on composite beams incorporating bolted shear connectors and discovered that their structural behavior was comparable to that of beams with welded stud connectors based on experimental findings. Ban et al.^18^, Pathirana et al.^19,20^, and Henderson et al.^21^ examined the performance of composite beams utilizing blind bolts as shear connectors and concluded that they effectively facilitate composite action, comparable to welded studs. Additionally, their studies suggested that blind bolts positively influence the long-term behavior of composite beams under sustained loading conditions. Liu et al.^22^ developed a design formula for high-strength friction-grip bolts based on tests of composite beams with geopolymer precast slabs (≥ 40 MPa). Results showed superior ductility over traditional connectors and demonstrated that, under service loads, these composite beams could be disassembled, allowing for reuse of individual components. Király and Dunai^23^ developed and examined novel bolted shear connectors with embedded bolts and threaded rods, tailored for industrial application. Push-out tests demonstrated their adequate strength and ductility, aligning with Eurocode 4 standards. Findings confirm their suitability for practical implementation, meeting the objectives of the research and development project. Additionally, Hamoda et al.^24^ experimentally and numerically analyzed steel I-beams with and without high-strength bolted connectors embedded in normal and Steel Fiber-Reinforced concrete (SFRC). The study examined composite action using studs or bolts, emphasizing bolted connectors for small sections. Push-out tests evaluated concrete type, surface roughness, bolt quantity, and stirrups. Results highlighted their impact on load-slip behavior and failure mode, demonstrating SFRC’s benefits in cracking resistance and capacity enhancement. A formula was proposed to estimate ultimate shear capacity for push-out failure mode.

Numerous studies have effectively identified the failure modes of concrete-steel connections. Zhang et al.^10^ conducted eleven push-out tests, revealing that local crushing occurred beneath the connector shank in specimens made with concrete grades below 50 MPa. Yang et al.^25^ conducted an experimental study on bolted connectors, where failure occurred by shearing off in a concrete slab with a thickness of 500 mm and bolts of 70 mm height. They tested four types of connectors and found that increasing the bolt diameter resulted in a higher peak slip, influenced by the bolt’s grade, height, and diameter. In another study, Weng et al.^26^ performed experiments to investigate shear splitting failure in composite concrete-encased steel beams. The results showed prominent horizontal cracks at the interface between the steel flange and concrete, signifying shear splitting failure in multiple beams. Additionally, many studies suggest that splitting cracks in concrete are more likely when the steel section is similar in width to the composite section, when reinforcement is lacking, or when there is an insufficient number of nails to transfer forces properly^27,28^.

There is an increasing trend toward incorporating high-strength concrete in composite structures to minimize spalling in concrete slabs^29^. Researching bolted steel–concrete connections with high-strength concrete is critical given limited studies and the lack of design codes. Comparative analysis with traditional concrete can provide engineers with the necessary insights to implement high-strength concrete in bridges and high-rise buildings effectively and safely. Li An and Krister Cederwall^30^ are among the few researchers who explored this area, conducting experimental investigations on the shear bearing strength of normal and high-strength concrete with welded studs under push-out loading. The results showed that the shear bearing load of the stud increased by 34% as concrete strength rose from 30 to 81 MPa. Normal concrete specimens exhibited more ductile behavior in load-slip curves post-peak load than those made from high-strength concrete. In addition, Shim et al.^31^ performed push-out tests on shear studs placed in high-strength and fiber-reinforced concrete, materials frequently used in high-rise constructions. These tests evaluated the shear strength and load-slip characteristics of the connectors. The results were compared with finite element model predictions and current design code standards.

Numerous studies have been conducted to examine various parameters in push-out tests. Jiang et al.^32^ examined the behavior of partially encased composite beams under negative bending, emphasizing longitudinal reinforcement ratios. Results showed enhanced concrete-steel bonding from the checkered steel pattern and delayed flange yielding with web encasement, reducing local buckling. They further analyzed the impact of shear connector quantity on shear resistance, offering strategies to improve composite beam performance and structural integrity while addressing failure prevention. Push-out tests assessed how varying concrete cover (90–170 mm) impacts the bond-slip behavior in steel-reinforced concrete specimens with I-shaped steel sections. While the steel section dimensions varied, concrete cube size (500 × 300 × 660 mm) and reinforcement were constant. Results showed initial and ultimate bond stress improved by 36.9% and 28% when concrete cover increased from 90 to 140 mm. Detailed descriptions of splitting failure around steel sections were provided. The study builds on prior research, including Shim et al.^33^, Lin et al.^34^, and Kozma et al.^35^, which explored push-out tests of shear connectors in conventional concrete.

To date, despite extensive research on steel–concrete composite sections, the behavior of tubular steel columns (TSC) with and without bolted shear connectors embedded in normal concrete have been rarely conducted. To the best of the authors’ knowledge, only one study has investigated steel I-beams with and without high strength bolted connectors embedded in normal concrete and SFRC^24^. As a result, the primary objective of this study is to experimentally and numerically analyze the behavior of tubular steel columns (TSC) with and without bolted shear studs embedded in normal concrete. The selection criteria for the tested specimens were based on practical structural applications and previous research on shear connectors in composite structures. The variables, including demountable shear studs and reinforcement, were chosen to evaluate their influence on load transfer mechanisms. The finite element model (FEM) was developed and validated against experimental results to ensure accuracy. The parametric study explored key factors affecting TSC performance, selected based on their structural significance and influence on shear capacity, such as TSC section thickness (t), TSC tensile strength, concrete compressive strength (${f}_{c}$), shear stud height, and bolt diameter.

## Experimental program

### Specimen’s details

The study involved three composite box tubular steel column specimens constructed with normal concrete (NC). The investigation focused on two variables: the presence of demountable shear studs and the inclusion of reinforcement. Detailed specifications of the specimens are presented in Table 1.

**Table 1:** Description of push-out test specimens.

Specimen ID	Demountable shear stud condition	Reinforcement condition
S_N_	No	No
S_NB_	With	No
S_NBR_	WITH	With

Nomenclatures of subscripts: N is normal concrete, B indicates the specimen with shear connectors, and R is reinforcement.

The push-out specimen consisted of a tubular steel column (TSC) completely encased in concrete. In each of the three specimens, the TSC was embedded 100 mm into the concrete block, with an additional 100 mm extending above the top of the concrete cube. The TSC was uniformly embedded 100 mm into the concrete block for all three specimens, with a 100 mm portion extending above the surface of the concrete cube. The 100 mm embedded length of the TSC within the concrete block meets the necessary requirements of the Egyptian Code^36^ to ensure adequate protection for the connectors within the concrete slab. The Egyptian Code of Practice for Steel Construction and Bridges provides clear guidelines for concrete cover around shear connectors, specifying a minimum lateral cover of 50 mm and a top cover of at least 20 mm. Furthermore, a 100 × 100 × 100 mm blue foam was placed beneath the TSC to create an empty space. This void enabled the examination of the bond between the TSC surface and the surrounding concrete without any interference. For all specimens, the concrete block was dimensioned at 250 × 250 × 200 mm. These dimensions were also selected to adhere to the Egyptian code^36^. The 250 mm side was also chosen to effectively observe any splitting cracks that may develop in the concrete.

**Fig. 1:**
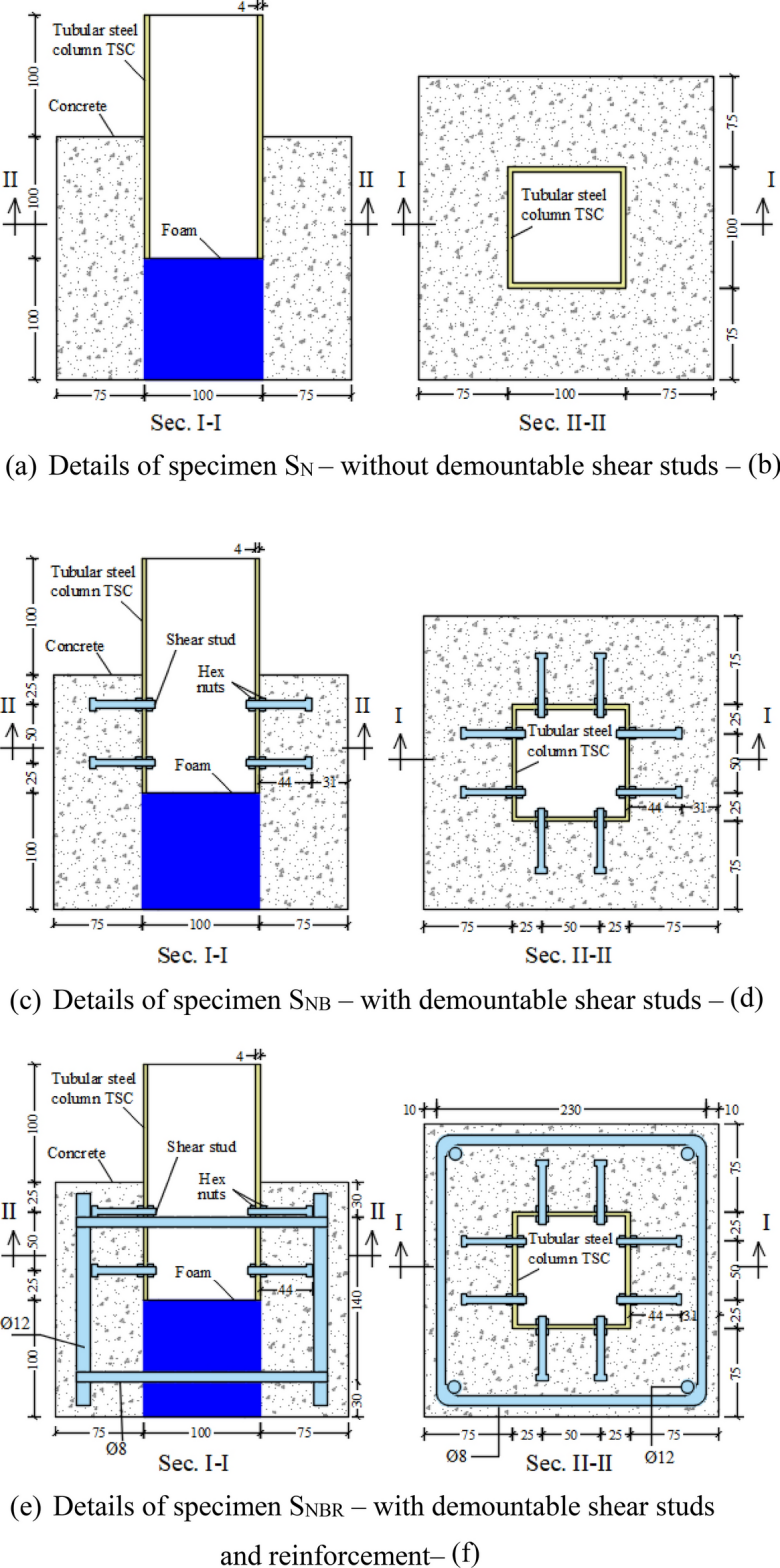
Details of all the tested specimens.

Figure 1 displays the details of all tested specimens. Figure 1a illustrates specimen S_N_, which was constructed without demountable shear studs to investigate the pure bonding behavior at the interface between the tubular steel column (TSC) and the concrete. Figure 1b illustrates specimen S_NB_, which features demountable shear studs consisting of 16 bolts. The selection and installation of the bolts adhered to the specifications outlined in the Egyptian code^36^. The bolt was selected and installed according to recommendations of the Egyptian code^42^ as followed: (1) height of the shear stud (h) must not be less than four time the bolt diameter 4Ø (4 × 6 mm = 24 mm) where h was selected to be 44 mm bigger than 24 mm, (2) nominal diameter of the stud head shall not be less than 1.5Ø where hex head of 10 mm was selected higher than 1.5Ø = 1.5 × 6 = 9 mm, (3) stud diameter Ø shall not exceed two times wed thickness of TSC where Ø was chosen to be 6 mm smaller than 2 × 4 mm = 8 mm, (4) maximum spacing between the bolts shall not exceed 600 mm, three times slab thickness (3 × 75 mm = 225 mm) and four times stud height 4h (4 × 44 mm = 176 mm) so that the stud pitch was selected 50 mm, (5) concrete cover after the stud head must bigger than 20 mm so it taken 31 mm.

This specimen was designed to investigate the impact of incorporating demountable shear studs. Additionally, Fig. 1c showed the details of the reinforced specimen (S_NBR_), which included demountable shear studs similar to those in specimen S_NB_. Vertical reinforcement was provided by four high-tensile steel (HTS) rebars with a diameter of 12 mm, while horizontal reinforcement consisted of two closed mild-steel stirrups with a diameter of 8 mm. The clear concrete cover was maintained at 10 mm. The lower stirrup was positioned 30 mm above the base of the concrete cube, and the upper stirrup was placed 30 mm below the concrete surface. This specimen was designed to evaluate the influence of existing reinforcement.

### Material properties

#### Concrete mix

The research utilized a single type of concrete mix: normal concrete (NC). The NC was composed of fine aggregate (sand), coarse aggregate (graded crushed basalt dolomite), 42.5 grade Portland cement, a superplasticizer, and water^37^. As detailed in Table 2, the mix proportions were designed with a ratio of 1:2.17:4.3 for cement, fine aggregate, and coarse aggregate, respectively, and a water-to-cement (w/c) ratio of 0.5. To evaluate the concrete’s performance, compressive strength tests were conducted on cylinders cast simultaneously with the tested specimens and cured under the same conditions. The compressive strength was determined by averaging the results from three 150 × 150 × 150 mm concrete cubes^38^. The normal concrete (NC) mix demonstrated an average compressive strength of 30 N/mm^2^.

**Table 2:** Compositions of utilized concrete.

Concrete mix type	Cement ($\text{kg}/{\text{m}}^{3}$)	Water $(\text{kg}/{\text{m}}^{3})$	Fine aggregate ($\text{kg}/{\text{m}}^{3}$)	Coarse aggregate ($\text{kg}/{\text{m}}^{3}$)	Super plasticizer ($\text{kg}/{\text{m}}^{3}$)	Water/cement (%)
NC	300	150	650	1290	12	0.50

#### Reinforcing rebar

**Fig. 2:**
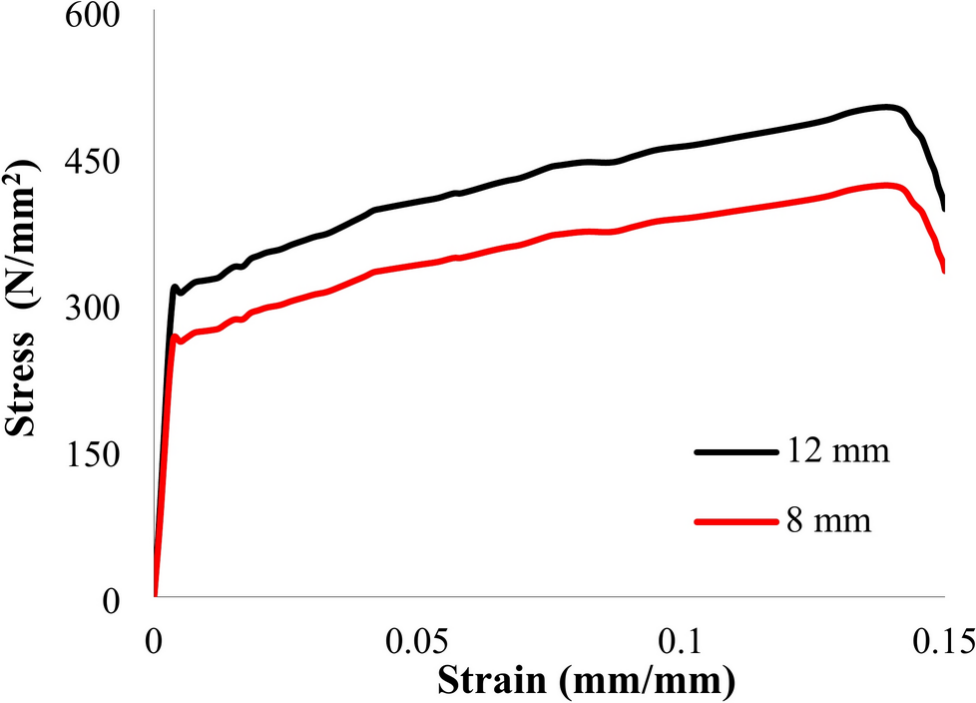
Stress–strain curves of the reinforcing bars.

Uniaxial tensile tests were performed on both the vertical and transverse reinforcing elements, with the results summarized in Table 3. The Ø12 mm reinforcement bars exhibited yield and ultimate tensile strengths of 320 MPa and 500 MPa, respectively. The Ø8 mm bars had yield and ultimate tensile strengths of 255 MPa and 420 MPa. The stress–strain curves for both types of reinforcing bars are presented in Fig. 2. By comparing the yield and ultimate stresses in Table 3 with the values specified in the Egyptian Code^36^, a partial alignment with the code is observed. The yield and ultimate stresses for normal mild steel (NMS) are 240 MPa and 350 MPa, respectively, while for high tensile steel (HTS), they are 360 MPa and 520 MPa.

**Table 3:** Properties of reinforcing rebars.

Type	Bar	Surface condition	Use	Poisson’s ratio	Elastic modulus (GPa)	Yield stress (MPa)	Ultimate stress (MPa)
NMS	8 mm	Smooth	Stirrups	0.3	200	255	420
HTS	12 mm	Deformed	Vertical	0.3	200	320	500

#### Steel box column and bolted shear connector

**Fig. 3:**
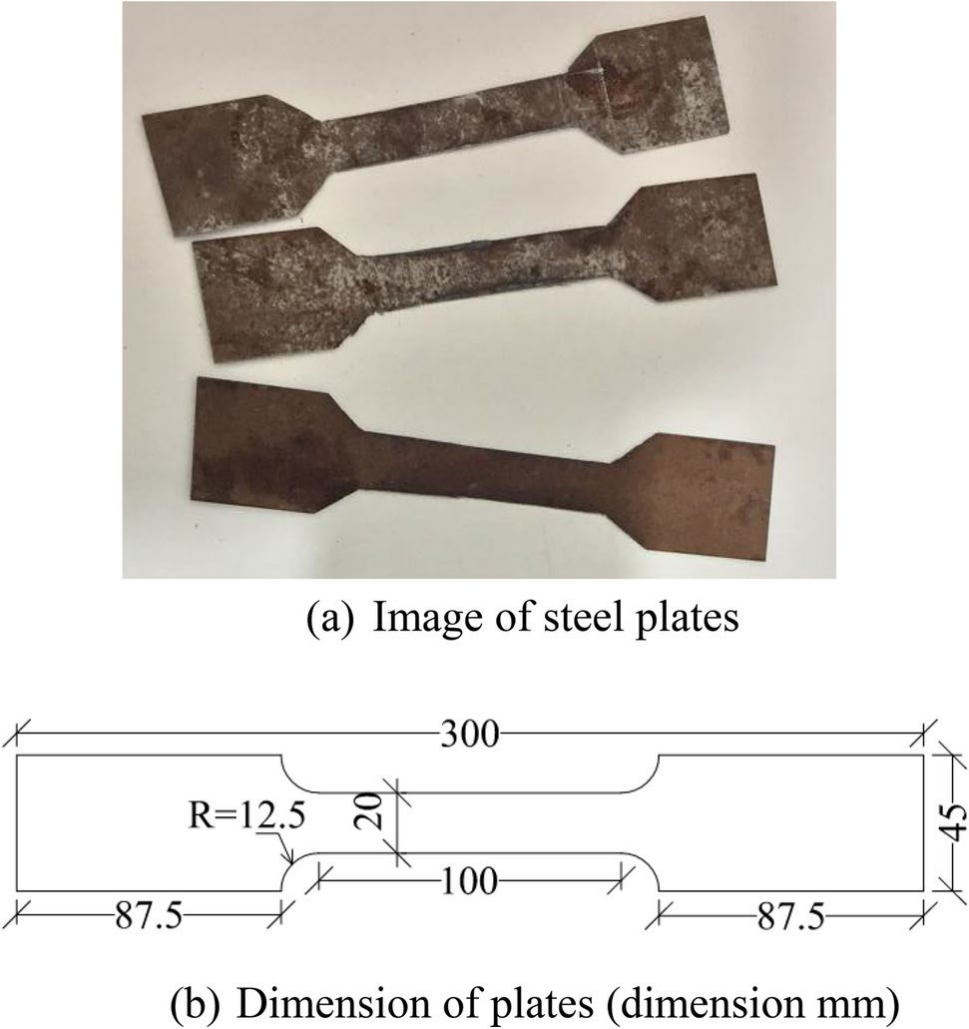
Steel plates taken from tubular steel column.

**Fig. 4:**
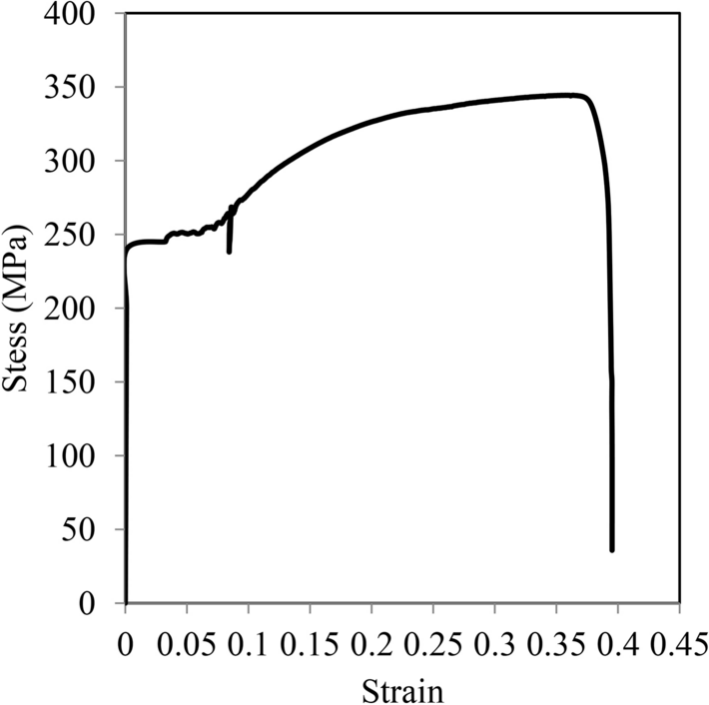
Stress strain relationship of tubular steel box section.

The steel box tubular section used in the tests had an external square cross-section of 100 mm and a wall thickness of 4 mm. To determine its mechanical properties, tensile tests were conducted on three plates extracted from the steel box, as shown in Fig. 3. In compliance with ASTM standard^39^, three plates were fabricated for testing. To minimize the risk of local failure at the ends, two enlarged sections, each measuring 8.75 cm in length, were incorporated. The plates had a total length of 30 cm and a width of 4.5 cm, as depicted in Fig. 3b. The strain rate is typically controlled within a range of 0.005–0.05 mm/mm/min. The resulting stress–strain curve is shown in Fig. 4. The average yield strength was 245 MPa, the ultimate strength was 336 MPa, with an average elongation of 39% and an elasticity modulus of 198 GPa.

**Fig. 5:**
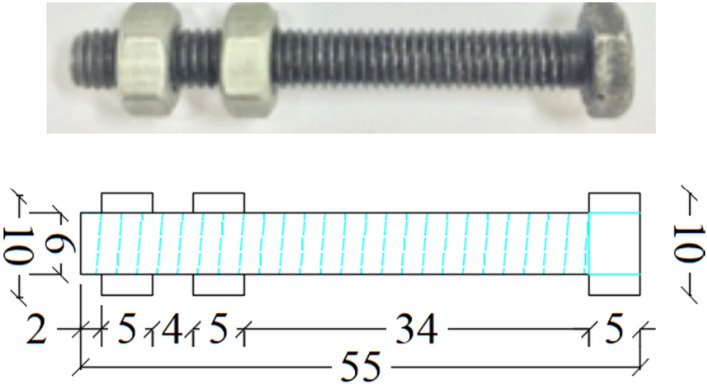
Shape and details of the used high strength bolt (dim. in mm).

A high-strength friction-grip bolt with a grade of 8.8 was utilized in this study, featuring a hexagonal head. The shape and specifications of the bolt are shown in Fig. 5. The bolt’s diameter was 6 mm, and its total length was 55 mm, with a shank height of 50 mm. The threaded portion matched the shank length. Two nuts, each with a 10 mm diameter, were used to secure the bolt in the web of the steel box tubular section. According to the bolt manufacturer, the bolt’s proof strength, yield strength, and ultimate tensile strength were 580 MPa, 640 MPa, and 800 MPa, respectively.

### Procedure for specimen preparation

**Fig. 6:**
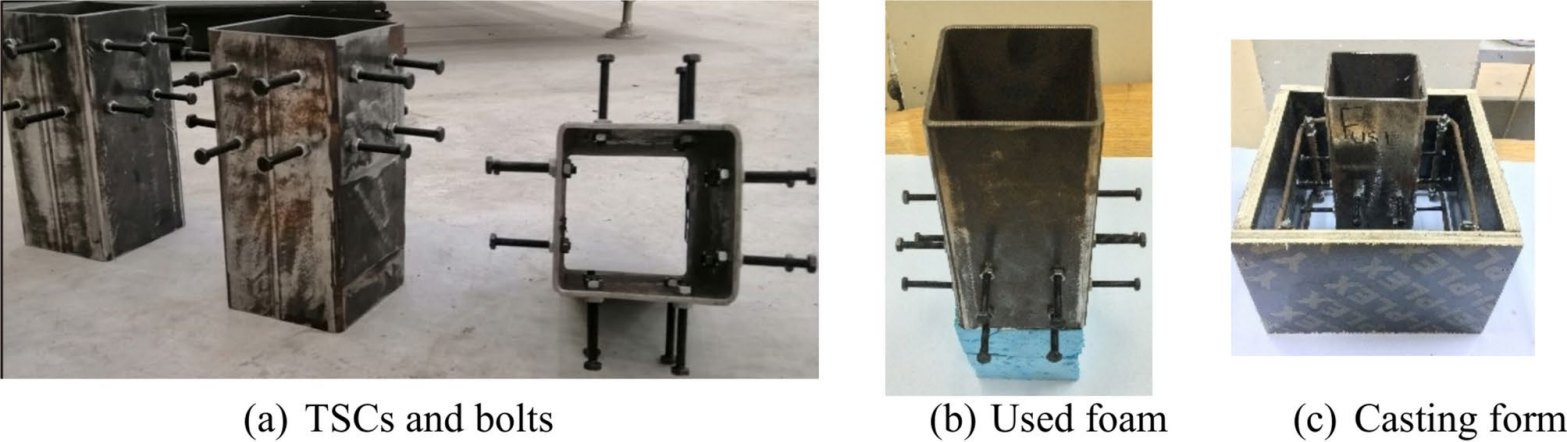
Procedure for specimen preparation.

Tubular steel columns, 200 mm in length, were prepared with flattened ends to ensure uniform load distribution. Sixteen 8 mm holes were drilled to accommodate bolts, which were securely fastened, as shown in Fig. 6a. A foam cube was adhered beneath the TSC for stability. The final specimen is shown in Fig. 6b. Additionally, a separate wooden mold (see Fig. 6c) was crafted for each specimen and coated with a moisture-resistant layer to prevent the absorption of water from the concrete mix. High-strength pre-tensioned bolts were employed, where the nut was tightened against the web surface of the steel box tubular section. This tightening process created a pre-tensioning force within the bolt. A wrench was used to securely fasten the bolt and ensure proper tensioning.

### Push out test setup

**Fig. 7:**
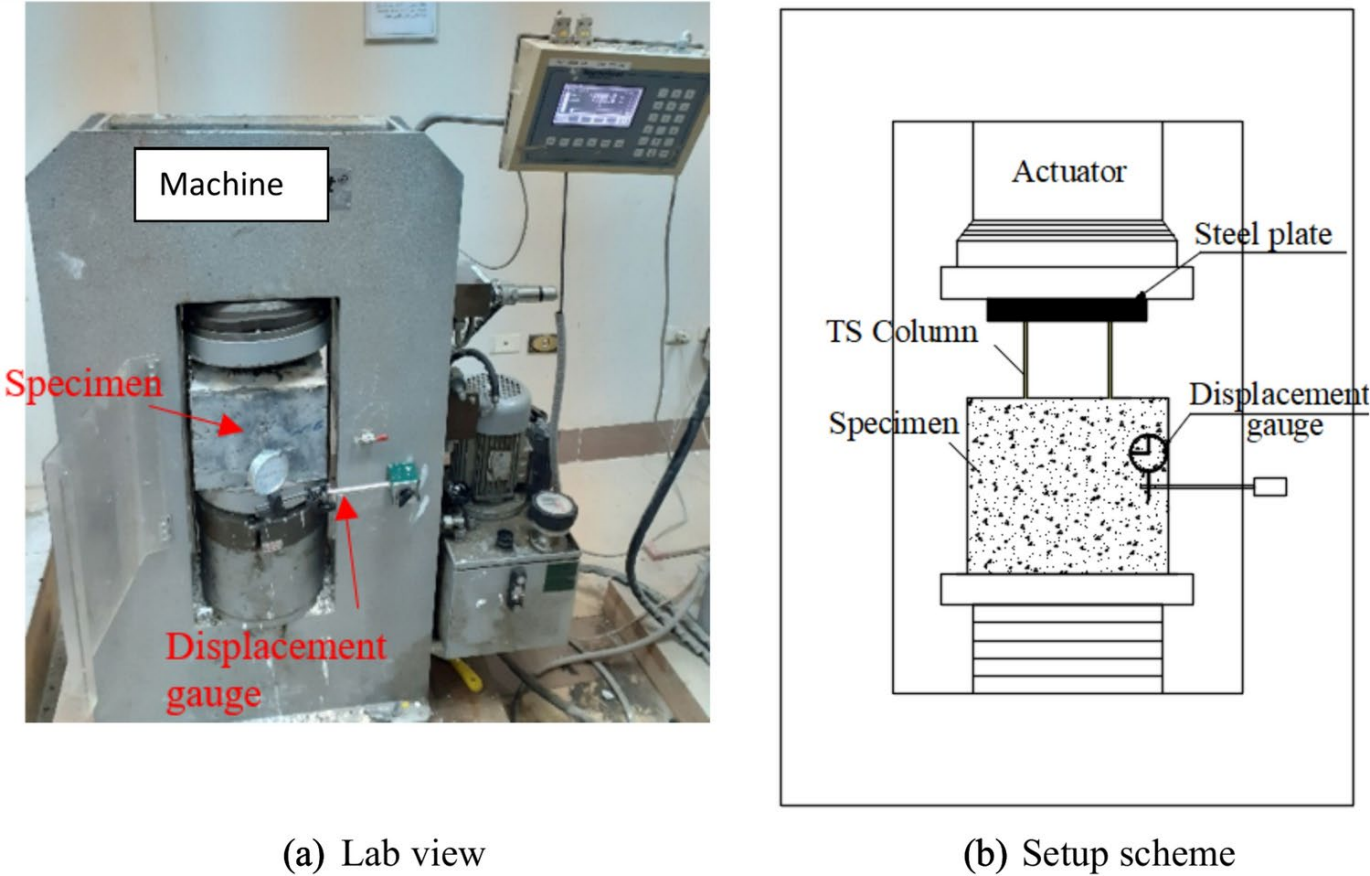
Push out test setup.

The push-out tests were conducted in accordance with the Egyptian Code^36^ using a uniaxial compression machine having a 2000 kN capacity, as depicted in Fig. 7. A rigid steel plate was positioned on top of the TSC to guarantee uniform distribution of the applied load across all shear connectors. The load was applied directly along the axis at the center of the specimen, with a precision of 0.1 kN. A force-controlled loading method was employed, with a constant rate of 125 N/s for all specimens. The slip displacement of the TSC during loading was recorded with a mechanical dial gauge, offering a precision of 0.01 mm.

## Results of tests and discussion

This section details the findings from the push out test, focusing on critical factors such as failure patterns, Load-slip relationship, peak load (${P}_{U}$), slip (${\delta }_{U}$), and Shear stiffness (K). Table 4 offers a detailed summary of the results, presenting the peak load (Pu), peak slip (δu), and shear stiffness (k). The shear stiffness (k) is determined from the slope of the load–displacement curve within its initial elastic region.

**Table 4:** Results of the bolt specimens.

Specimen ID	Peak load P_u_ (kN)	Peak slip δ_u_ (mm)	Shear stiffness		
			0.5 P_u_ (kN)	Corresponding slip (mm)	k (kN/mm)
S_N_	71.6	26.6	35.8	0.12	298.3
S_NB_	225.4	1.90	112.7	0.32	352.2
S_NBR_	226	3.05	113	0.27	418.5

### Failure patterns

**Fig. 8:**
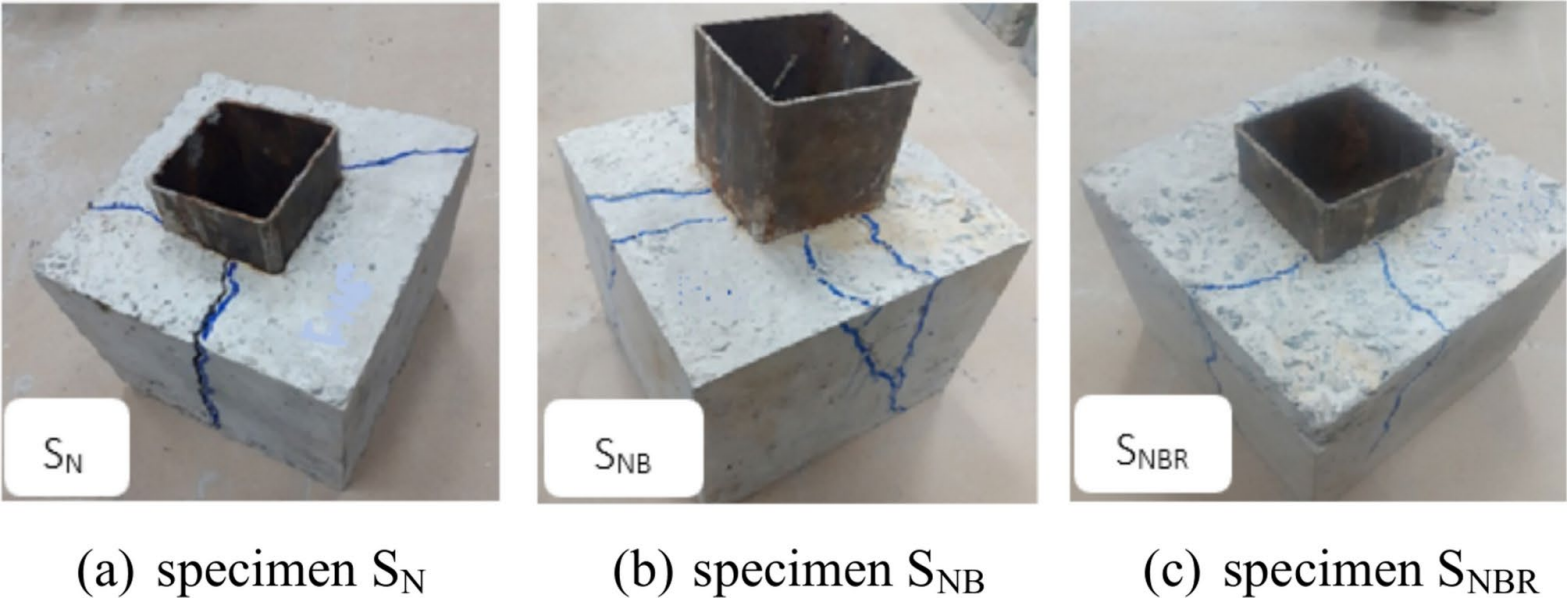
Failure modes of all the tested specimens.

Figure 8a presents the crack patterns observed in the non-bolted specimen. Specimen S_N_ exhibited a failure mode involving the splitting and obvious debonding of concrete within the cube. For the bolted specimens, a distinct sound was heard during testing, indicating a fracture in the connector bolt. The crack propagation followed this sequence: it originated near the connectors, spread upward and downward, and eventually extended horizontally across the top and soffit of the concrete cube, as shown in Fig. 8b,c.

**Fig. 9:**
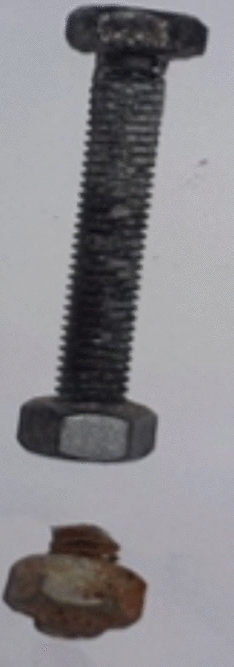
Bolt fracture at TSC-concrete interface.

**Fig. 10:**
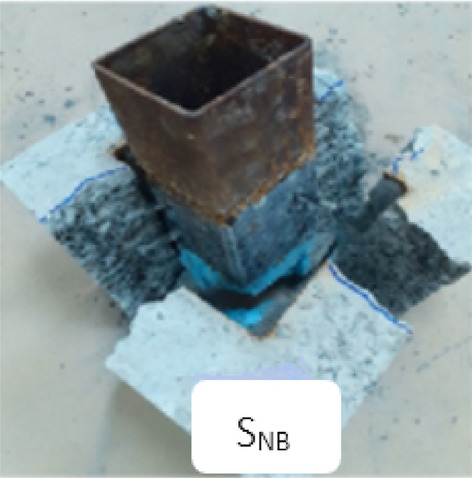
Concrete splitting and accompanied with crooked connectors.

Additionally, for the bolted specimens, failure of the bolt resulted from a stud shank fracture at the TSC-concrete interface, as shown in Fig. 9. Figure 10 illustrates the bent connector configuration and localized concrete crushing beneath the bolt.

### Load-slip relationship

**Fig. 11:**
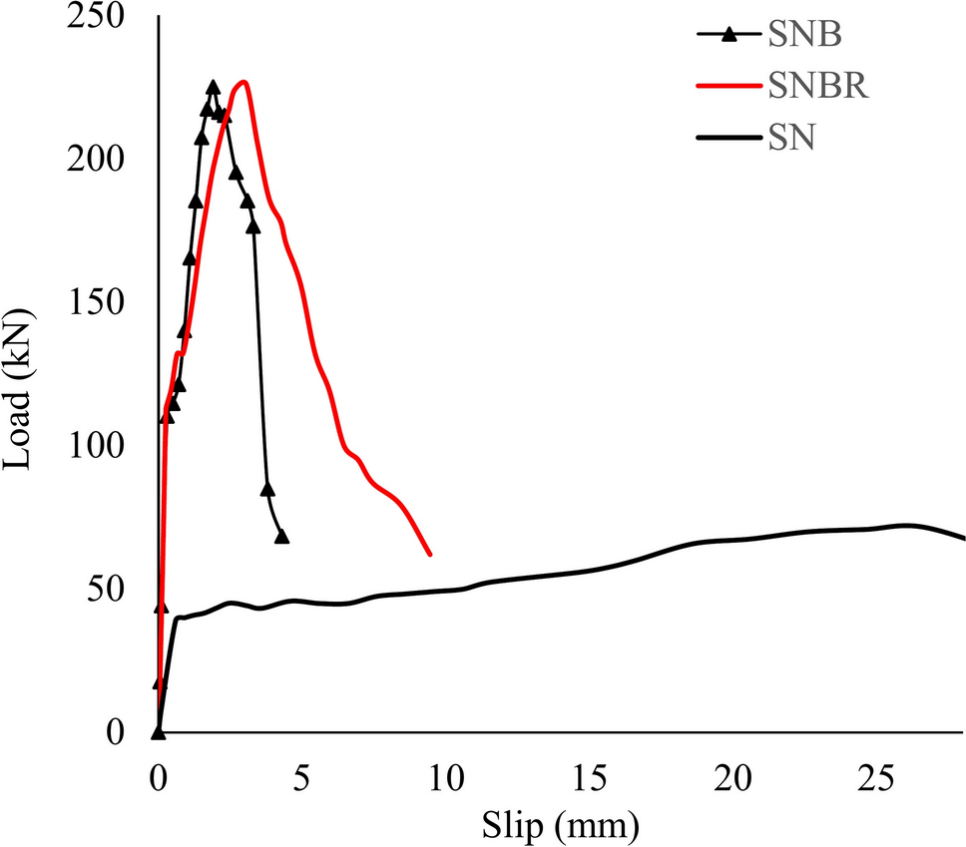
Load-slip curves of the specimens.

The load-slip curves for the tested specimens are shown in Fig. 11, which illustrates five distinct phases: the initial bond at the steel–concrete interface, the bolt shank slipping phase (represented by a flat section), followed by the linear, nonlinear, and decline phases. In this study, the slope of the curve was defined as the shear stiffness. The connector slipping phase was clearly evident in all specimens. It was observed that the shear stiffness decreased compared to the stage before the bolt shank slipping. The initial stage continued until 40–50% of the failure load. As the load increased, the load-slip curves entered a linear stage, which then transitioned into a nonlinear stage characterized by reduced stiffness, eventually reaching the peak load. In the final stage, the shear capacity of the specimens gradually decreased after reaching the peak, with the shear connectors progressively failing one by one.

### Peak load, slip, and shear stiffness

Table 4 summarizes the experimental data for the specimens’ containing bolts and reinforcement, highlighting their peak load (P_u_), peak slip (δ_u_), and shear stiffness (k). Shear stiffness for each specimen was determined by dividing half of the peak load (P_u_) by the associated slip (δ). The peak load values for each specimen are provided in Table 4. The peak loads for specimens S_NB_ and S_NBR_ were 225.4 kN and 226 kN, respectively, indicating that the inclusion of reinforcement had negligible effect on their shear bearing capacity under pushout loading conditions. Additionally, the slip at the peak load of each specimen was defined as the peak slip (δu). As shown in Table 4, δu for specimens S_NB_ and S_NBR_ were 1.90 mm and 3.05 mm, respectively. The results indicate that the reinforcement increased the peak slip of S_NBR_ by 37.7% compared to S_NB_. The increase in slip for the S_NBR_ specimen after 0.5 P_u_ compared to the S_NB_ specimen can be attributed to the presence of reinforcement, which enhances ductility and deformation capacity. While reinforcement may not significantly affect the peak load**,** it improves the bonding and load transfer mechanism between the concrete and steel components, allowing for greater slip before failure.

Furthermore, the shear stiffness (k) values for S_NB_ and S_NBR_ were found to be 352.2 kN/mm and 418.5 kN/mm, respectively. This also indicates a positive effect of reinforcement on shear stiffness, with S_NBR_ exhibiting an 18.7% increase in k compared to S_NB_. The P_u_ of sample S_N_ was the least (71.6 kN) compared to that of sample S_NB_. When the shear studs were performed in the sample S_N_B, the P_u_ increased by 217% over sample S_N_ with no bolts indicating that the inclusion of bolts had clear effect on their shear bearing capacity under push out loading conditions.

## Numerical simulation

Extensive finite element simulations were conducted using ABAQUS/Explicit software to model the push-out tests of all the tested specimens. The complete finite element model was used to address initial uncertainties related to analysis techniques, boundary conditions, mesh design, material models, and overall model behavior, ensuring an accurate representation of the push-out test specimens. In-depth finite element models of bolts and headed studs were used to analyze their performance as shear connectors, with a primary focus on assessing differences in shear resistance. Once the numerical results aligned well with the experimental data, a parametric study was conducted using the detailed finite element models to explore the effects of factors such as the shear connector height, concrete grades, and other relevant variables on the shear bearing capacity under pushout loading conditions.

### Geometry, boundary conditions and loading

**Fig.12:**
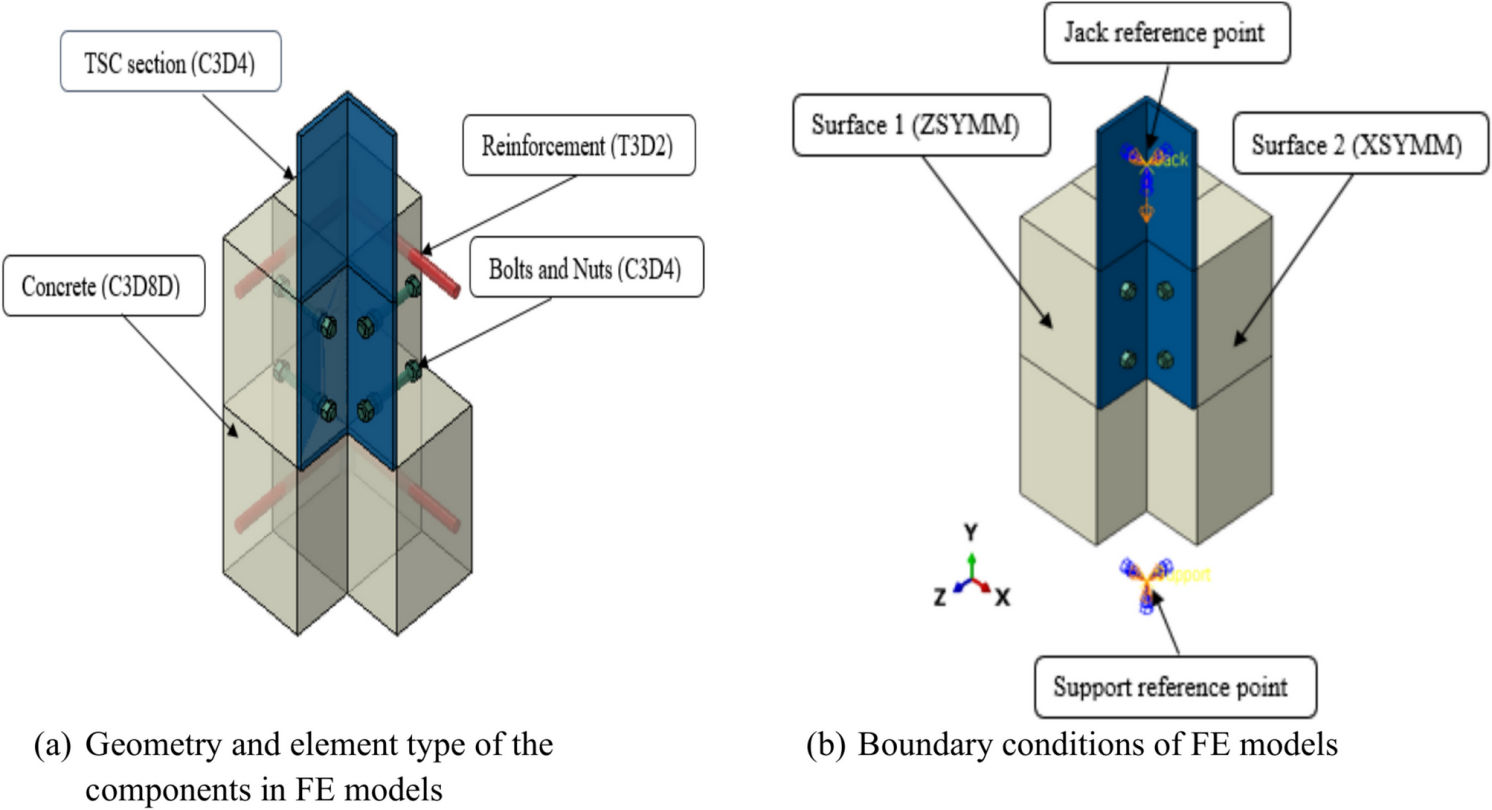
FEM models geometry and boundary conditions.

The comprehensive FEA model incorporated all elements used in the push-out tests, including the concrete, steel section, bolts, nuts, and reinforcement bars, as shown in Fig. 12a. The bolts and nuts were accurately modeled to capture the precise geometry of their heads and threads, while the reinforcement bars were represented as distinct solid components embedded within the concrete, as illustrated in the Fig. 12a.

Given the symmetry of the specimens, only one-quarter of each was modeled in ABAQUS. As shown in Fig. 12b, symmetry boundary conditions were applied to Surfaces 1 and 2, which correspond to the concrete slab and the web of the steel section, respectively. Therefore, all nodes on Surface 1 were fixed against displacement in the Z direction and rotation in the X and Y directions. Similarly, all nodes on Surface 2 were restricted from translation in the X direction and rotation in the Y and Z directions. The support at the bottom of the concrete slab was fixed to prevent movement in all three directions. It is important to mention that the load area and abutment surface were restricted by two reference points (refer to Fig. 12b). The “Jack” and “Support” reference points were utilized for applying the loads and setting the support conditions in the models.

### Analysis method, mesh and contact modeling

The explicit dynamic solver in ABAQUS was employed to conduct a quasi-static analysis of the FE specimens. This approach is effective for addressing nonlinear problems involving material damage, large deformations, and intricate contact interactions^40^. To generate the mesh for the FE models, three-dimensional 8-node linear elements with reduced integration (C3D8R) were utilized of concrete. Due to the complex geometry of the steel section, bolts, and nuts, tetrahedral elements (C3D4) were employed. It is crucial to highlight that shell elements were inadequate for modeling the steel section, as the interaction between bolts and the steel section plays a critical role in determining the connection’s ultimate strength. To represent the reinforcing bars, 2-node linear truss elements (T3D2) were utilized.

**Fig. 13:**
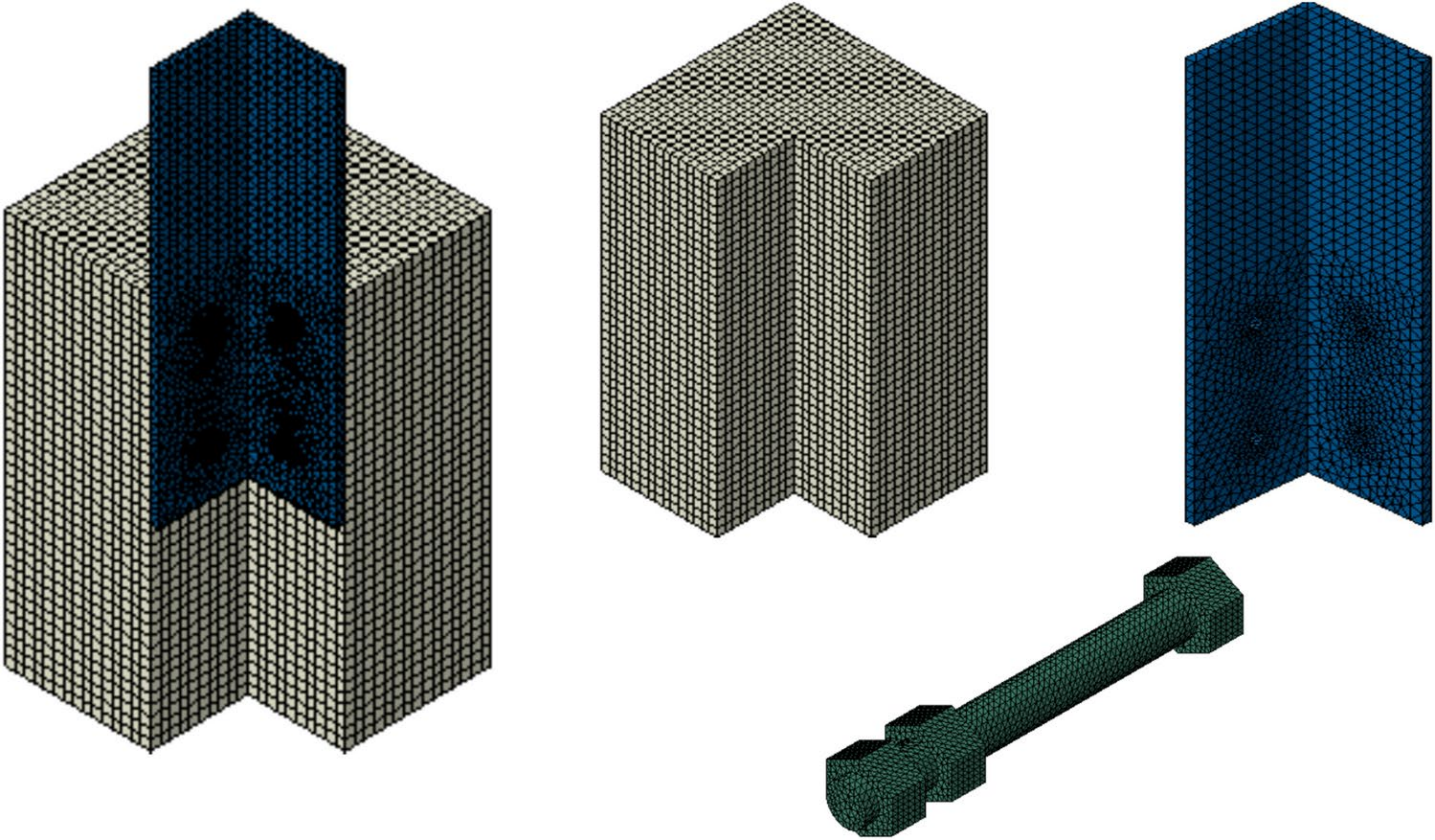
Mesh configuration of the components in FE models.

The mesh size across different parts was optimized to ensure minimal impact on the final results while maintaining an efficient analysis time. Each component was assigned a different mesh size, with finer elements focused on areas of stress concentration. The global mesh sizes were set to 2 mm for the bolts and nuts, 5 mm for the steel section, and 10 mm for the concrete, as shown Fig. 13. For the interaction, surface-to-surface contacts were applied between the various parts of the specimens. ABAQUS defines contact properties in two ways: normal behavior and tangential behavior. For normal behavior, a “hard” contact model was applied, where pressure was directly related to the closure, and separation was permitted once contact was broken. The tangential behavior was represented using a penalty friction model, with a friction coefficient of 0.14 assigned between the concrete and the steel section, as specified in^17^. Also, a friction coefficient of 0.5 was calibrated between the set of bolts and nuts and the steel section. Furthermore, the embedded method was utilized to model the bond between the reinforcing bars and the concrete.

### Material modeling

#### Concrete model

The model incorporates the material properties of normal concrete within ABAQUS. ABAQUS offers two primary material modeling techniques: the smeared cracking approach and the concrete damaged plasticity (CDP) method^41–46^. Each approach is capable of accurately representing the behavior of plain concrete. The Concrete Damage Plasticity (CDP) model was selected for the push-out test simulation due to its capability to accurately capture the nonlinear behavior of concrete under combined compression and tension, including damage evolution. Since the primary objective of this study is to investigate shear behavior, the CDP model is appropriate as it accounts for concrete cracking, crushing, and stiffness degradation, which are critical in shear-dominated responses. Additionally, the model effectively represents the interaction between concrete and steel, ensuring realistic simulation of bond-slip mechanisms and load transfer^47–49^.

Numerous researchers have proposed equations to model concrete behavior under uniaxial compression. However, certain formulations, including those by Mander et al.^50^ and Yong et al.^51^, have failed to accurately capture the full stress–strain characteristics of concrete. To overcome this shortcoming, Saenz^52^ stress–strain equations are utilized to provide a detailed representation of concrete behavior under uniaxial compression, as outlined in Eqs. (1)–(7).

1 $${\sigma }_{c}=\frac{{E}_{c}{\varepsilon }_{c}}{1+\left(R+{R}_{E}-2\right)\frac{{\varepsilon }_{c}}{{\varepsilon }_{o}}-\left(2R-1\right){\left(\frac{{\varepsilon }_{c}}{{\varepsilon }_{o}}\right)}^{2}+R{\left(\frac{{\varepsilon }_{c}}{{\varepsilon }_{o}}\right)}^{3}}$$

2 $$E_{c} = 4700\sqrt {f_{c}{\prime} } \quad {\text{for}}\,{\text{normal}}\,{\text{strength}}\,{\text{concrete}}\,[53]$$

3 $$R= \frac{{R}_{E}({R}_{\sigma }-1)}{{\left({R}_{\varepsilon }-1\right)}^{2}}-\frac{1}{{R}_{\varepsilon }}$$

4 $${R}_{E}=\frac{{E}_{C}}{{E}_{O}}$$

5 $${R}_{\sigma }=\frac{{f}_{c}{\prime}}{{\sigma }_{f}}$$

6 $${R}_{\varepsilon }=\frac{{\varepsilon }_{f}}{{\varepsilon }_{o}}$$

7 $${E}_{O}=\frac{{f}_{c}{\prime}}{{\varepsilon }_{O}}$$

where ${\sigma }_{c}$ is concrete compressive stress (MPa), ${E}_{c}$ is Modulus of elasticity of concrete (MPa), ${E}_{O}$ is Secant modulus of concrete (MPa), ${f}_{c}{\prime}$ is Maximum compressive strength of concrete (MPa), ${\varepsilon }_{c}$ is Compression strain; ${\varepsilon }_{o}:$ Strain corresponding to ${f}_{c}{\prime}$ which approximately equals 0.0025 as reported by Hu and Schnobrich^54^, ${\varepsilon }_{f}$ is Maximum strain, ${\sigma }_{f}$ is Stress at maximum strain (MPa), R is Ratio relation, ${R}_{E}$ is Modular ratio, ${R}_{\sigma }$ is Stress ratio, which is equal 4 as reported by Hu and Schnobrich^54^, ${R}_{\varepsilon }$ is Strain ratio, which is equal 4 as reported by Hu and Schnobrich^54^.

**Fig. 14:**
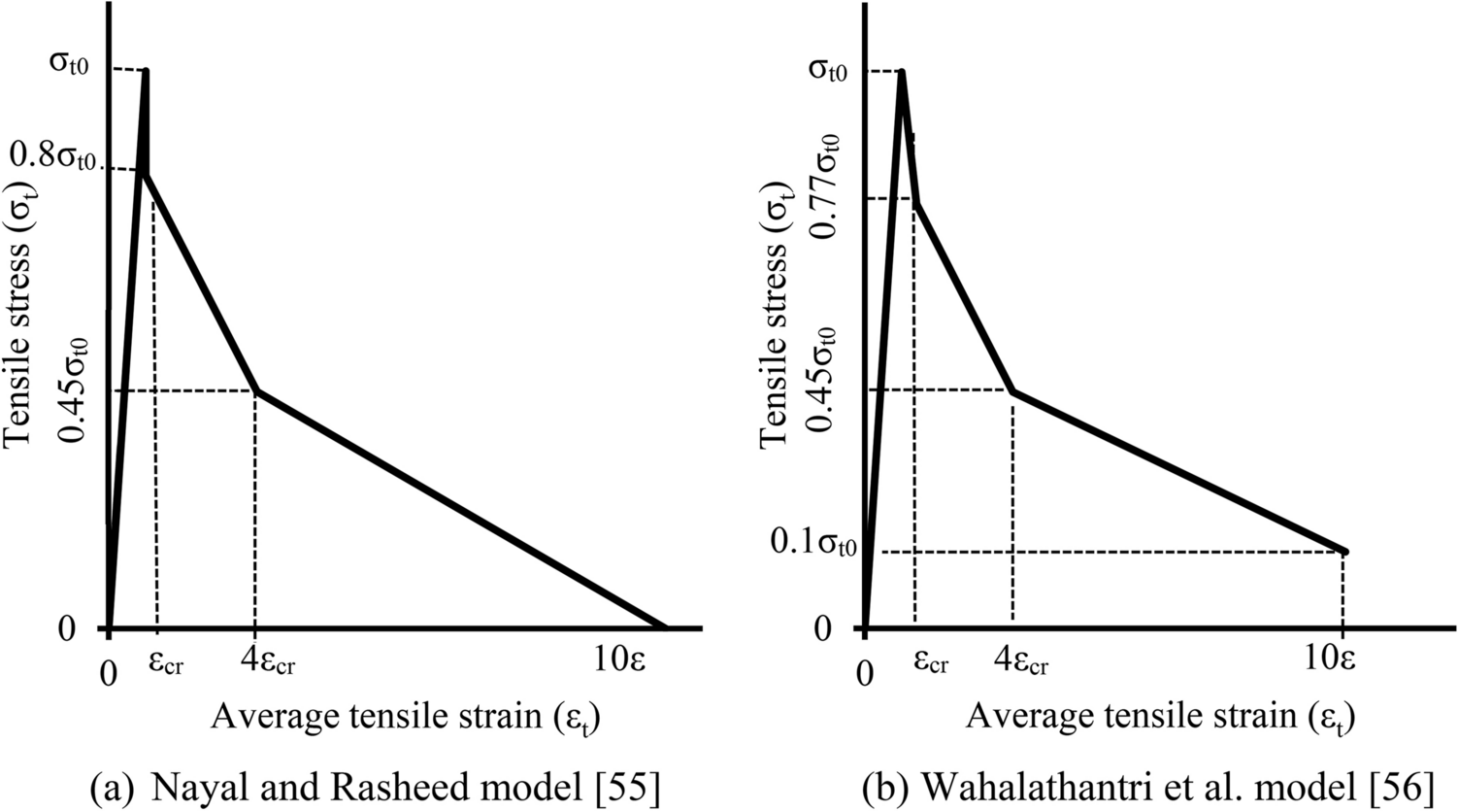
Tension model of concrete.

The tensile stress–strain curve for concrete used in the model is based on Nayal and Rasheed’s formulation^55^, later refined by Wahalathantri et al.^56^, as shown in Fig. 14.

**Fig. 15:**
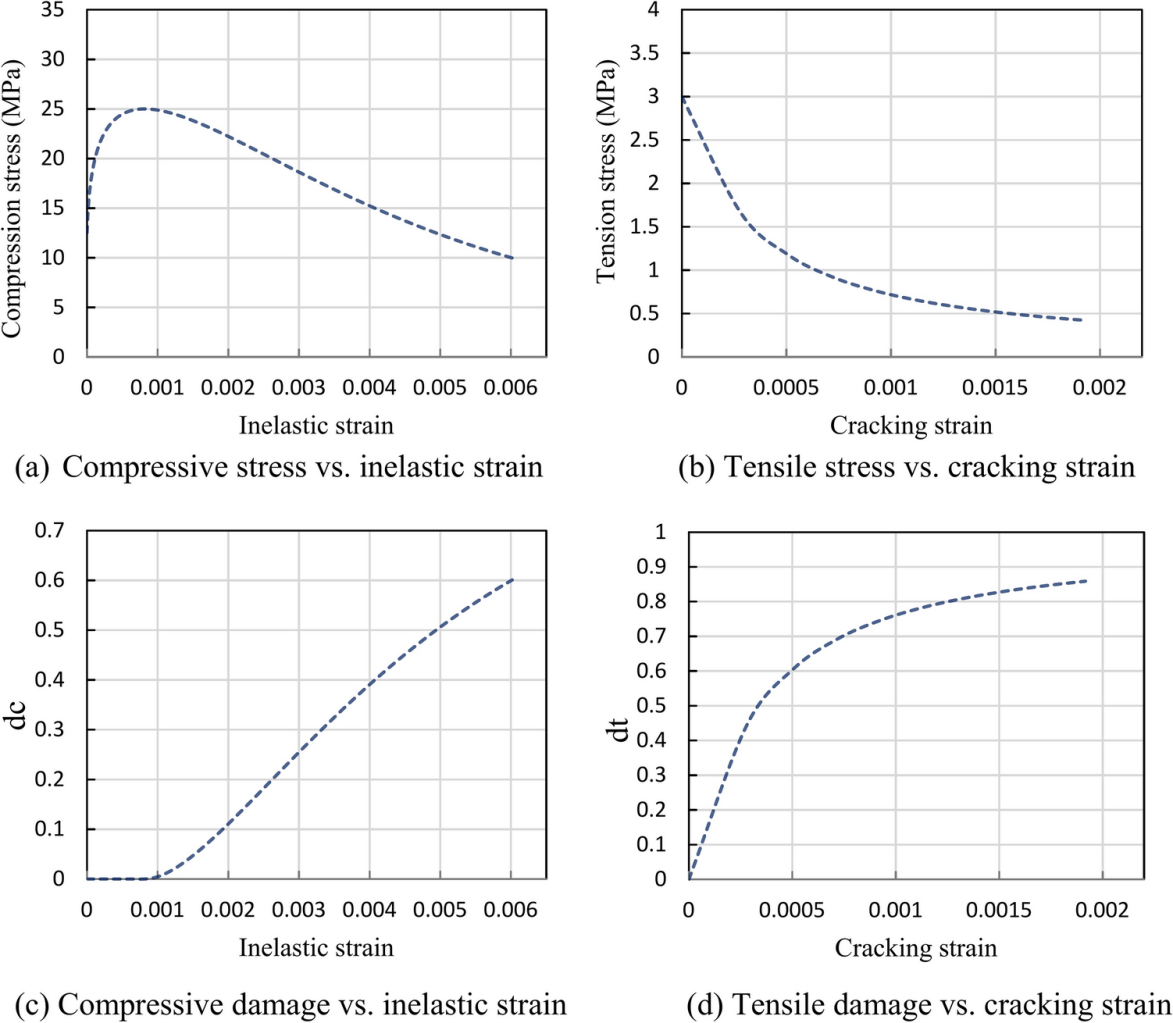
Concrete damage-plasticity model parameters in ABAQUS^59^.

Figure 15 presents critical information required to define concrete material properties in ABAQUS. Figure 15a shows the uniaxial compression stress-inelastic strain relationship, while Fig. 15b illustrates tension stress versus cracking strain. Figure 15c highlights the correlation between the compression damage parameter and inelastic strain, and Fig. 15d depicts the tension damage parameter variation with cracking strain. Additionally, the plasticity parameters were defined following ABAQUS guidelines^57^, with a flow potential eccentricity ε = 0.1 and a biaxial-to-uniaxial compressive strength ratio (${f}_{bo}/{f}_{co}$) of 1.16. The dilation angle (ψ = 38°) was calibrated through iteration to align with push-out test results, closely matching Jankowiak and Lodigowski’s^58^ recommendation (ψ = 38°)^31^, as summarized in Table 5.

**Table 5:** Parameters of damage-plasticity model.

Parameter	Value
Dilation angle (ψ)	38°
Eccentricity (ε)	0.10
Viscosity relaxation parameter (μ)	0.008
Ratio of biaxial to uniaxial compressive yield stresses (${f}_{bo}/{f}_{co}$)	1.16
Ratio of the second stress invariant on the tensile to the compressive meridian (${K}_{c}$)	0.667

#### Steel materials

The bolt, headed stud, and steel section materials were modeled using isotropic plasticity^60^, characterized by an initial elastic modulus E_0_ = 210 GPa and a Poisson’s ratio ν = 0.3. To simulate failure mechanisms and element deletion, progressive damage models in ABAQUS were applied. Both ductile and shear damage models were implemented for the bolt and headed stud materials, whereas the steel section material was modeled with ductile damage alone.

The criteria for damage initiation and the corresponding evolution rules were derived based on the progressive damage models outlined in Ref.^61^. To establish the damage initiation criteria independently of strain rate, the equivalent plastic strain at damage onset (${\overline{\varepsilon }}_{0}^{pl}$) must be defined as a function of stress triaxiality (θ). Trattnig et al.^62^ conducted an experimental analysis on austenitic steels, establishing the following relationship between these variables:

8 $${\overline{\varepsilon }}_{f}^{pl}=\alpha . \text{exp}(-\beta . \theta )$$

where, ${\overline{\varepsilon }}_{f}^{pl}$ represents the equivalent plastic strain at fracture, while α and β are material-specific constants. Rice and Tracey^63^ introduced a similar fracture curve, demonstrating an exponential relationship between void growth rate and triaxiality. Under uniaxial tension with θ = 1/3, the ratio ${\overline{\varepsilon }}_{f}^{pl}/{\varepsilon }_{f}^{pl}$ is determined using the following equation:

9 $${\varepsilon }_{f}^{pl}=\alpha . \text{exp}(-\beta . 1/3)$$

10 $${\overline{\varepsilon }}_{f}^{pl}/{\varepsilon }_{f}^{pl}=\text{exp}[-\beta . \left(\theta -1/3\right)]$$

By assuming that the ratios of equivalent uniaxial strain at damage initiation and fracture are equal (${\overline{\varepsilon }}_{f}^{pl}/{\varepsilon }_{f}^{pl}={\overline{\varepsilon }}_{0}^{pl}/{\varepsilon }_{0}^{pl}$), and using the material constant β = 1.5 as suggested by Rice and Tracey^63^, the uniaxial plastic strain at the onset of damage can be calculated using Eq. (11).

11 $${\overline{\varepsilon }}_{0}^{pl}(\theta )={\varepsilon }_{n}^{pl} .\text{exp}[-1.5 \left(\theta -1/3\right)]$$

**Fig. 16:**
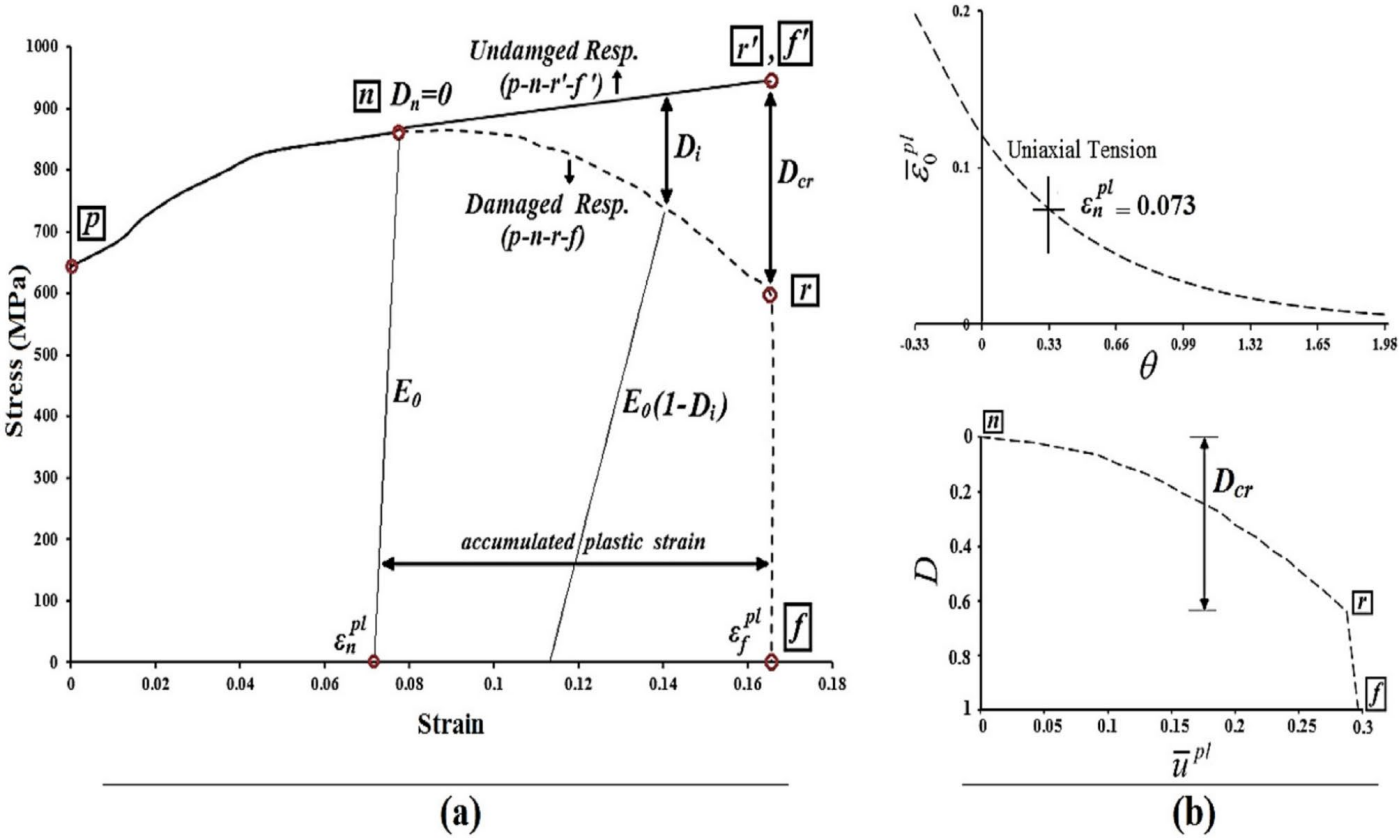
Required curves for steel materials: (**a**) plasticity curves, (**b**) damage curves^17^.

The damage parameter (D_i_) represents the dimensionless variation between the material’s behavior in its damaged and undamaged states. As illustrated in Fig. 16a, at the rupture point (r), the steel material reaches a critical damage threshold (D_cr_), which quickly leads to the fracture point (*f*), where there is a complete loss of stiffness. Lamaitre^64^ estimated that the critical damage parameter for most steels lies between 0.2 and 0.5. However, these values were derived under the assumption of a uniform damage parameter distribution across the cross-section. When accounting for the non-uniform distribution, the core of the cross-section experiences higher equivalent plastic strain, leading to increased values of the critical damage parameter. Bonora et al.^65^ reported that the actual values of D_cr_ in steel materials fall within the range of 0.55 to 0.65 (Fig. 16b). Therefore, in this study, the Eq. (12) proposed by Pavlović et al.^17^ was utilized to compute the value of the damage parameter.

12 $${D}_{i}=\left\{\begin{array}{c}(1-{\sigma }_{i}^{true}/\overline{{\sigma }_{i}}){\alpha }_{D} n\le i<r\\ 1 i=f\end{array}\right.$$

where ${\alpha }_{D}$ is the damage eccentricity factor.

To incorporate the damage evolution laws into the model, the damage parameter (${D}_{i}$) as a function of equivalent plastic displacement (${\overline{u} }_{i}^{pl}$) was adopted in tabular form. The value of ${\overline{u} }_{i}^{pl}$ is given by Eq. (13), where ${\overline{u} }_{f}^{pl}$ represents the total equivalent plastic displacement at the point of fracture. The parameter ${\overline{u} }_{f}^{pl}$ is determined by multiplying the element length (${l}_{e}$) by the cumulative plastic strain throughout the damage process^17^. Thus, Eq. (14) was applied to compute this parameter. According to Ref.^17^, ${\lambda }_{m}$ serves as a modification factor to account for the influence of mesh density and element type.

13 $${\overline{u} }_{i}^{pl}=\frac{{\overline{u} }_{f}^{pl}\left({\varepsilon }_{i}^{pl}-{\varepsilon }_{n}^{pl}\right)}{({\varepsilon }_{f}^{pl}-{\varepsilon }_{n}^{pl})} i\ge n$$

14 $${\overline{u} }_{f}^{pl}={\lambda }_{m }{l}_{e}({\varepsilon }_{f}^{pl}-{\varepsilon }_{n}^{pl})$$

In addition to the ductile damage model, a shear damage model was developed for the bolt material, as previously mentioned. Shear damage parameters are typically calibrated based on the target outcomes^17,66^. The damage initiation criterion defines the shear stress ratio and equivalent plastic strain, which were set at 1.7 and 0.2, respectively. Furthermore, damage evolution was modeled with displacement control, exponential softening, and multiplicative degradation, considering its interaction with ductile damage. The failure displacement and exponential parameter were calibrated to 0.4 mm and 0.7, respectively.

Given its lower significance, a linear elastic-perfectly plastic model with a yield strength was chosen for the reinforcing bars. Additionally, for all steel components, the elastic modulus was set to 210 GPa, and the Poisson’s ratio was fixed at 0.3.

### Numerical results

**Fig. 17:**
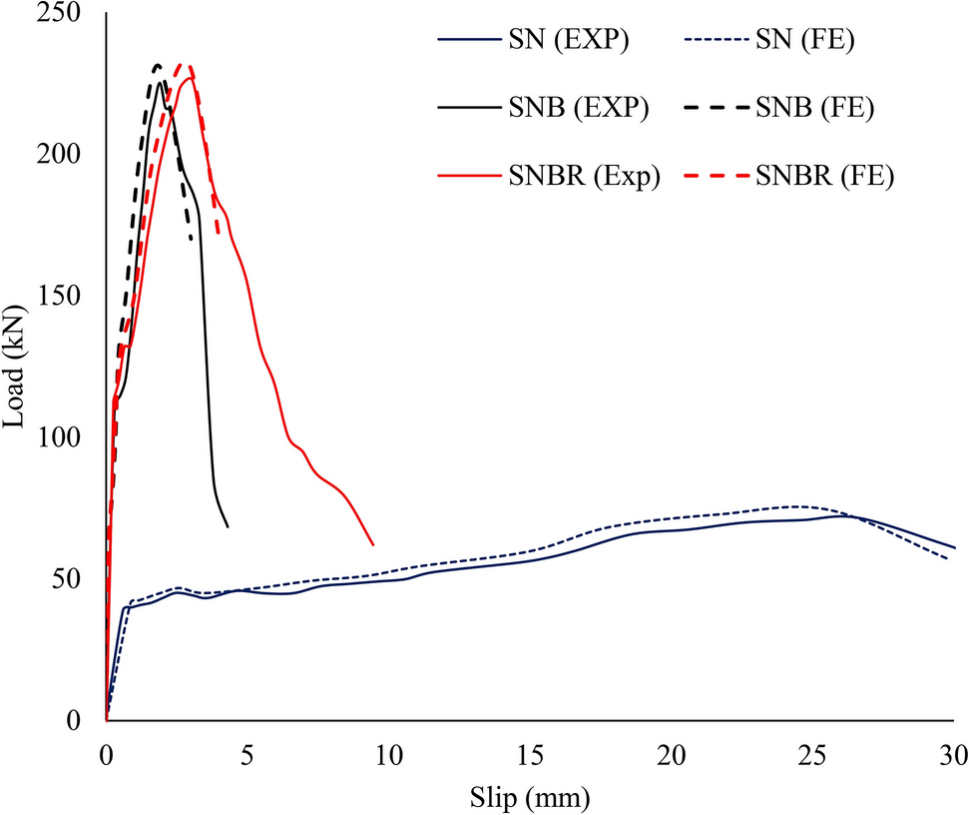
Experimental and numerical load-slip curves of the tested specimens.

**Fig. 18:**
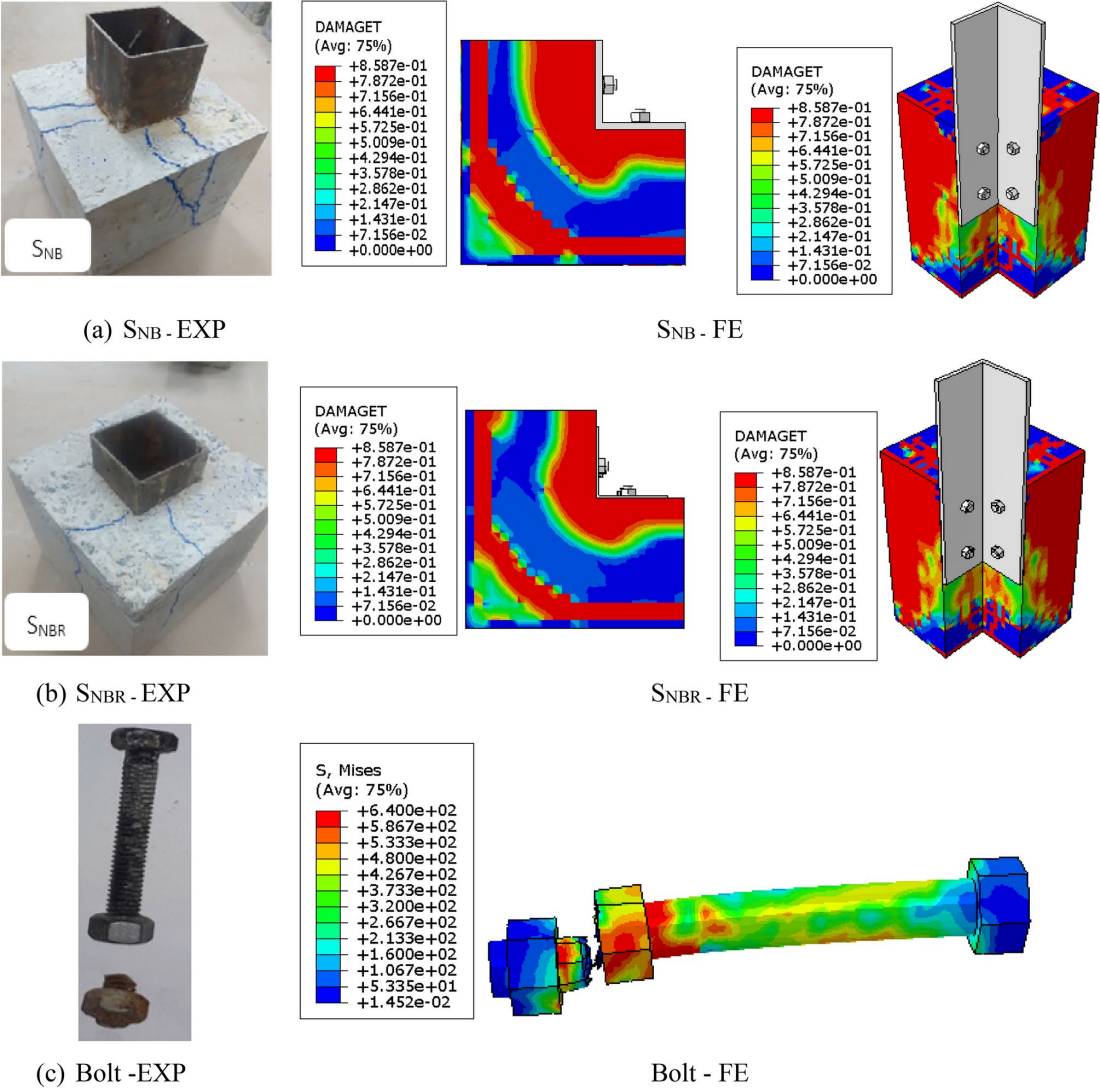
Comparison of experimental and numerical failure patterns.

The numerical results, particularly those concerning load-slip behaviour and failure patterns, were rigorously validated against experimental data, as presented in Figs. 17 and 18. Key values derived from the load-slip curves are detailed in Table 6. Figure 18 presents a comparison of the experimental and numerical failure patterns. In the numerical model, crack propagation followed this sequence: it began near the connectors, spread upward and downward, and ultimately extended horizontally across the top and bottom surfaces of the concrete cube for both S_NB_ and S_NBR_ specimens, as shown in Fig. 18a,b. Furthermore, the bolt failure was caused by a fracture of the stud shank at the TSC-concrete interface, as shown in Fig. 18c. As a result, the FEM demonstrate its proficiency in accurately capturing the effects of demountable shear studs and reinforcement on the cracking patterns and failure mechanisms observed in the tested specimens. Furthermore, the results highlight the model’s effectiveness in representing the impact of varying structural arrangements under the same applied loads.

**Table 6:** Evaluation of numerical versus experimental data.

Specimen ID	Peak load P_u_ (kN)			Peak slip δ_u_ (mm)		
	Exp	FE	FE/Exp	Exp	FE	FE/Exp
S_N_	71.6	73.8	1.030	26.6	25.7	0.97
S_NB_	225.4	230.5	1.023	1.90	1.907	1.004
S_NBR_	226	232	1.026	3.05	2.93	0.960
μ			1.026			0.978
SD			0.0028			0.019
CoV			0.0028			0.019

*Exp* Experimental, *FE* Finite element, *μ* Average deviation, *SD* Standard deviation, *CoV* Coefficient of variation.

Table 6 illustrates that at the ultimate stage, the average deviations (μ) were 1.026 for the ratio of finite element peak load to experimental peak load (${P}_{u,FE}/{P}_{u,Exp}$) and 0.978 for the ratio of finite element peak slip to experimental peak slip (${\delta }_{u,FE}/{\delta }_{u,Exp}$). The corresponding standard deviations (SD) were 0.0028 and 0.019, with coefficients of variation (CoV) of 0.0028 and 0.019. The FEM demonstrated a strong correlation with the experimental results.

## Parametric study

**Figure 19:**
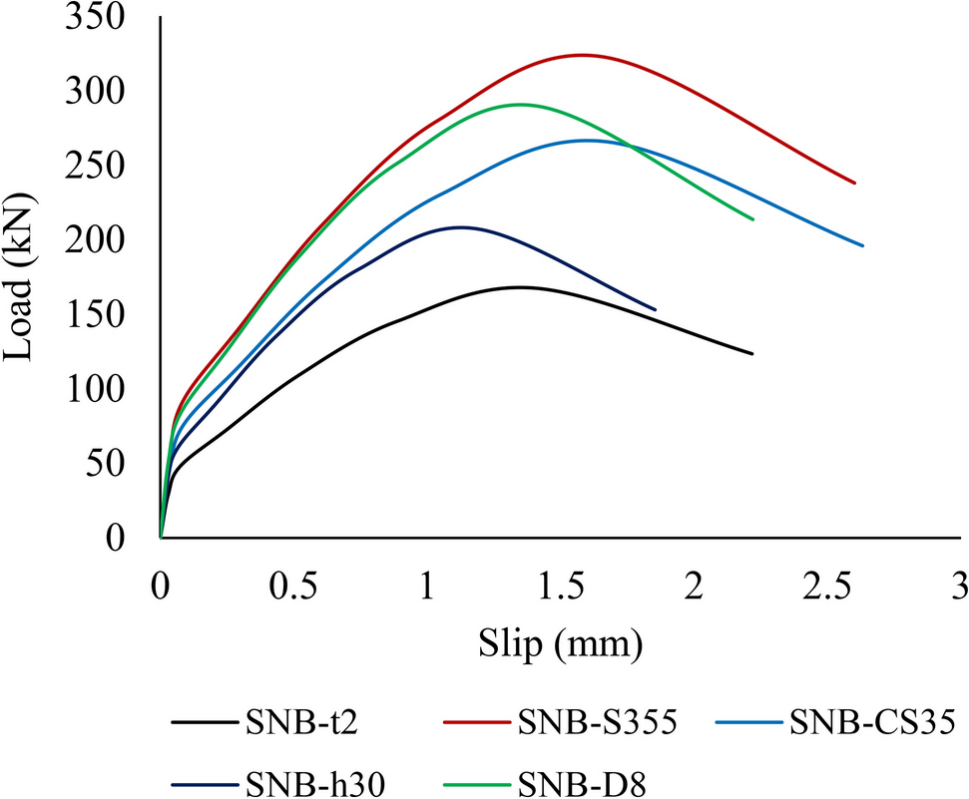
Load-slip curves of some specimens included in the parametric study.

A comprehensive parametric analysis was conducted using the validated models to evaluate the ultimate strength of bolted shear connectors. This study aimed to thoroughly examine the various factors influencing the ultimate capacity of these connectors. The analysis focused on five key variables: TSC section thickness (*t)*, TSC tensile strength, compressive strength of the concrete (${f}_{c}$), shear stud height, and bolt diameter. The S_NB_ specimen was utilized as the reference for conducting a comprehensive parametric analysis. Table 7 provides more details on the FE groups and their results for the parametric study. Additionally, Fig. 19 illustrates the load-slip behavior of some specimens included in the parametric study.

**Table 7:** Summary of the parametric study results.

Group	Specimen	TSC thickness (mm)	TSC tensile strength (MPa)	Concrete strength (MPa)	Shear stud height (mm)	Bolt diameter (mm)	Peak load (kN)	Peak slip (mm)
G1	S_NB_-t2	2	245	25	44	6	167.41	1.412
	S_NB_-t3	3					198.66	1.652
	S_NB_-t4	4					230.52	1.907
	S_NB_-t5	5					327.72	1.414
	S_NB_-t6	6					425.74	1.187
G2	S_NB_-S245	6	245	25	44	6	230.52	1.907
	S_NB_-S275		275				255.39	1.908
	S_NB_-S355		355				321.25	1.656
	S_NB_-S420		420				374.21	1.413
G3	S_NB_-CS25	6	245	25	44	6	230.52	1.907
	S_NB_-CS35			35			265.64	1.675
	S_NB_-CS45			45			276.24	1.605
	S_NB_-CS50			50			287.27	1.53
G4	S_NB_-h30	6	245	25	30	6	208.41	1.18
	S_NB_-h44				44		230.52	1.907
	S_NB_-h50				50		241.40	1.905
G5	S_NB_-D6	6	245	25	44	6	230.52	1.907
	S_NB_-D8					8	284.59	1.414
	S_NB_-D10					10	284.15	1.414
	S_NB_-D12					12	283.73	1.414

### TSC thickness (t)

**Fig. 20:**
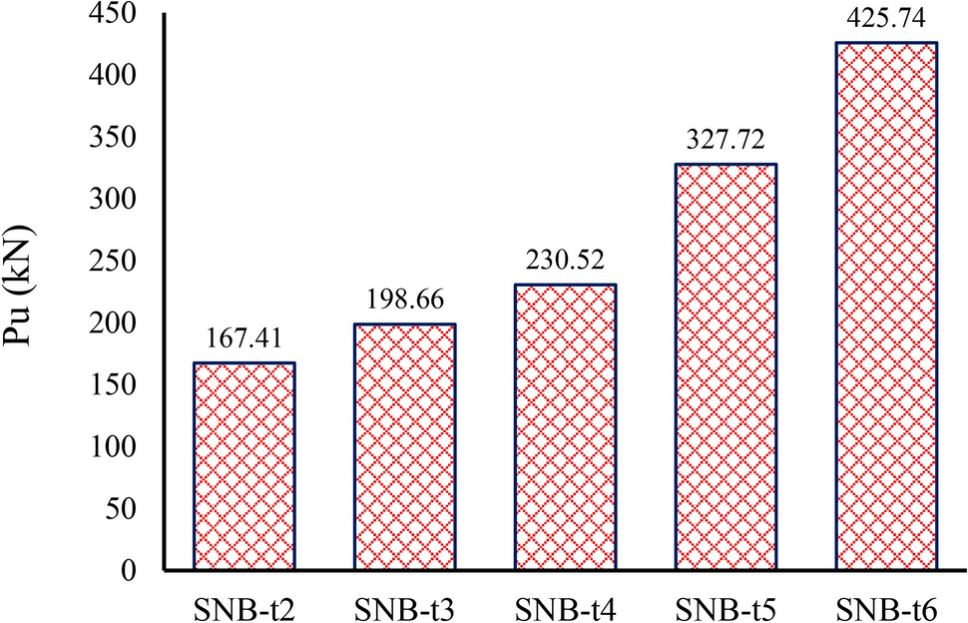
Effect of TSC thickness on the ultimate force per shear connectors.

For the parametric study, TSC thicknesses (t) of 2 mm, 3 mm, 4 mm, 5 mm, and 6 mm were selected for group G1, as detailed in Table 7. Figure 20 depicts the ultimate force per shear connector for specimens with the five TSC thicknesses. The results show that the peak loads (${P}_{u}$) for specimens with TSC thicknesses of 2 mm, 3 mm, 4 mm, 5 mm, and 6 mm are 167.41 kN, 198.66 kN, 230.52 kN, 327.72 kN, and 425.74 kN, respectively. An increase in TSC thickness results in a significant rise in peak load, with the peak load increasing by approximately 154.31% as the thickness grows from 2 mm (one-third of the bolt diameter) to 6 mm (bolt diameter). For specimens with 2 mm and 3 mm thicknesses, the primary failure mechanism was bearing within the TSC section, with no visible damage to the concrete slab or bolted connectors, highlighting the insufficiency of the thickness relative to the bolt diameter (6 mm). Given that the bolt diameter is 4 mm, and to prevent such failures, it is recommended that the thickness of the TSC section be greater than half the bolt diameter. This aligns with the requirements of the Egyptian code^36^. Additionally, the slip capacities for specimens with TSC thicknesses of 2 mm, 3 mm, 4 mm, 5 mm, and 6 mm are 1.412 mm, 1.652 mm, 1.907 mm, 1.414 mm, and 1.187 mm, respectively (see Table 7). This suggests that specimens with thicknesses greater than half the bolt diameter (SNB-t5 and SNB-t6) exhibit higher peak loads but smaller slip capacities, indicating lower ductility in these samples.

The variation in slip behavior for group G1 specimens can be attributed to the changing failure mechanisms as TSC thickness increases. Up to a thickness of 4 mm, both peak load and slip increase due to the enhanced load transfer capacity and improved confinement effect, which allow greater deformation before failure. However, for thicknesses of 5 mm and 6 mm, the specimens exhibit a more brittle failure mode. The increased TSC stiffness restricts deformation, leading to higher load-bearing capacity but lower slip capacity. This suggests a shift from a ductile failure mechanism to a more rigid behavior, reducing the ability of the specimen to undergo large deformations before failure.

### TSC tensile strength

**Fig. 21:**
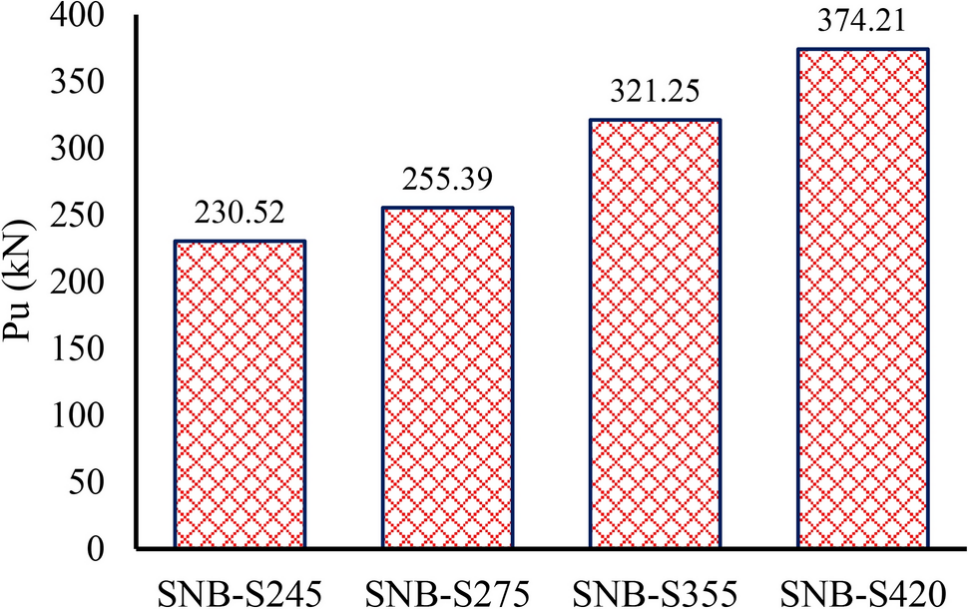
Effect of TSC tensile strength on the ultimate force per shear connectors.

In group G2 (Table 7), for the parametric analysis, three grades of structural steel—S275, S355, and S420—were selected in accordance with the European Code^67^. Their nominal stress–strain relationships were defined using the bilinear model with nonlinear hardening, as described by Yun and Gardner^68^. Figure 21 depicts the ultimate force per shear connector for specimens with the four TSC tensile strength. The findings reveal that the peak loads (${P}_{u}$) for the specimens with TSC tensile strengths of S245, S275, S355, and S420 are 230.52 kN, 255.39 kN, 321.25 kN, and 374.21 kN, respectively. As the tensile strength of the TSC increases, a noticeable rise in peak load is observed, increasing by about 62.33% as the material transitions from S245 to S420. It indicates the significant impact of TSC tensile strength on the ultimate force per shear connector. Additionally, the slip capacities for the specimens with TSC tensile strengths of S245, S275, S355, and S420 are 1.907 mm, 1.908 mm, 1.656 mm, and 1.413 mm, respectively (see Table 7). This also indicates that as the tensile strength of the TSC increases, the peak loads rise while the ductility decreases.

The observed trend in Group G2, where increasing the tensile strength of TSC results in higher peak loads but lower slip capacities, is primarily attributed to the mechanical behavior of high-strength steel and its interaction with concrete. As the tensile strength of TSC increases, the material’s ability to resist shear forces improves, leading to higher ultimate load capacities. This is because stronger TSC sections experience less deformation under load, enhancing load transfer efficiency between steel and concrete.

However, the decrease in slip capacity with increasing TSC tensile strength is due to the reduced ductility of higher-strength steel. While stronger TSC materials can withstand greater loads, they exhibit lower deformation before failure, making them more brittle compared to lower-strength steel. This reduced ductility limits the slip capacity, as less deformation occurs before the peak load is reached.

Additionally, the higher stiffness of stronger TSC sections results in a more rigid load transfer mechanism, restricting slip movement. In contrast, lower-strength TSC allows more deformation, leading to higher slip values. This behavior aligns with the general trade-off between strength and ductility in structural materials—higher strength enhances load-bearing capacity but reduces deformation capacity before failure.

### Compressive strength of the concrete

**Fig. 22:**
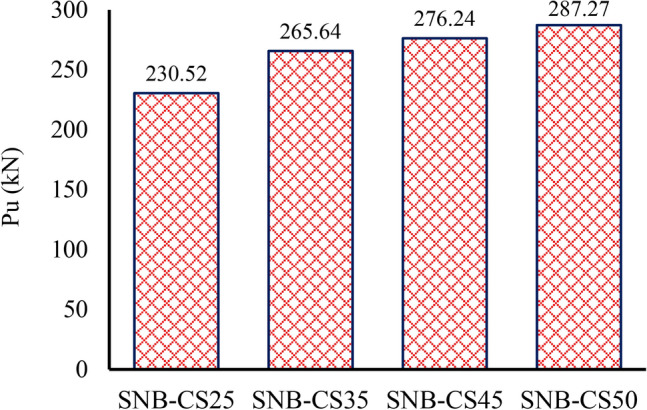
Effect of the concrete’s compressive strength (${f}_{c}$) on the ultimate force per shear connector.

For the parametric study, compressive strength of the concrete (${f}_{c}$) of 25 MPa, 35 MPa, 45 MPa, and 50 MPa were selected for group G3, as detailed in Table 7. Figure 22 depicts the ultimate force per shear connector for specimens with the four different concrete’s compressive strength. The data indicate that the peak loads (${P}_{u}$) for concrete compressive strengths (${f}_{c}$) of 25 MPa, 35 MPa, 45 MPa, and 50 MPa are 230.52 kN, 265.64 kN, 276.24 kN, and 287.27 kN, respectively. As the compressive strength of the concrete increases, there is a slight increase in peak load, with the ultimate peak load rising by approximately 24.6% as the compressive strength ranges from 25 to 50 MPa. Moreover, as the concrete’s compressive strength increases, the slip capacity reduces, dropping from 1.907 mm in specimen SNB-C25 to 1.53 mm in specimen SNB-C50, reflecting a decrease of 19.77%.

The observed trend in Group G3, where increasing concrete compressive strength leads to higher peak loads but lower slip capacities, can be attributed to the material behavior of concrete under load. As compressive strength increases, concrete exhibits greater resistance to deformation, enhancing the load-bearing capacity of the shear connectors. This results in higher peak loads as stronger concrete better confines the shear studs, improving load transfer efficiency.

However, the reduction in slip capacity with increasing compressive strength is due to the reduced ductility of higher-strength concrete. Stronger concrete is more brittle and less capable of accommodating deformation, leading to lower slip values before failure. This trend aligns with the well-known trade-off between strength and ductility in concrete materials—higher strength improves load resistance but reduces the ability to undergo large deformations before failure.

Additionally, the improved confinement of the shear studs in higher-strength concrete restricts their displacement, further contributing to the observed decrease in slip capacity.

### Shear stud height

**Fig. 23:**
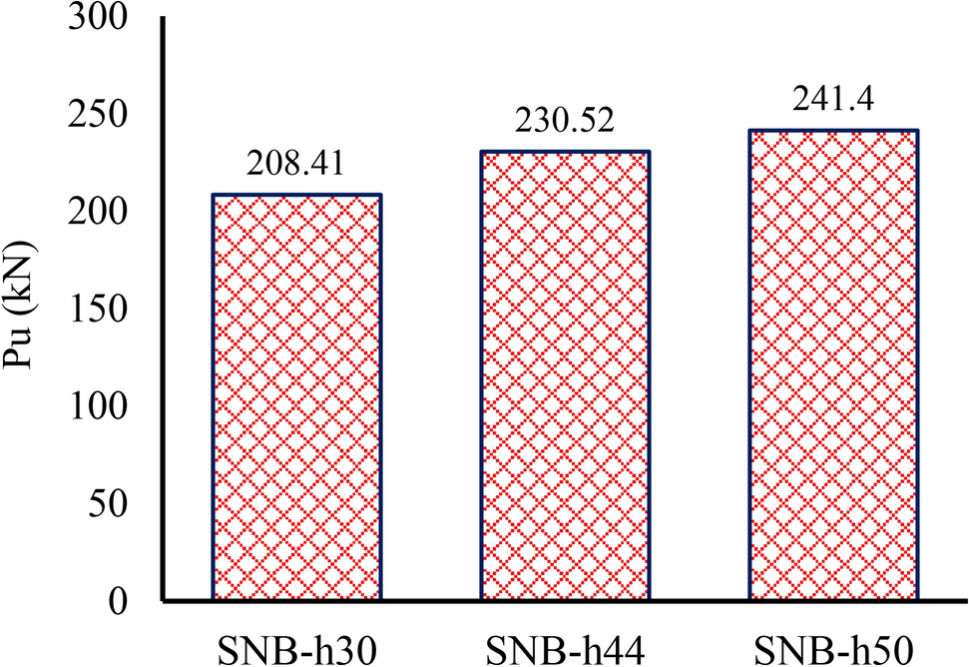
Effect of the height of the shear stud on the ultimate force per shear connector.

As shown in Fig. 23, three shear stud heights—30 mm, 44 mm, and 50 mm—were selected from Table 7 (group G4) to investigate the effect of shear stud height on the ultimate force applied to each shear connector. The results show that the peak loads (${P}_{u}$) for the specimens with shear stud heights of 30 mm, 44 mm, and 50 mm are 208.41 kN, 230.52 kN, and 241.40 kN, respectively. The results also show that as the bolt height increases, the peak load rise by 15.83% when the shear stud height changes from 30 mm (five time the bolt diameter) to 50 mm (eight time the bolt diameter). Additionally, the slip capacity increases as the shear stud height increases, from 1.18 mm for specimen SNB-h30 to 1.905 mm for specimen SNB-h50, representing a 61% increase.

Additionally, it can be noted that for Group G4 specimens, the increase in peak slip with peak load differs from other groups due to the effect of shear stud height on load transfer and failure behavior. Taller shear studs improve mechanical interlock and bonding with concrete, allowing greater deformation before failure and resulting in higher slip capacity. Conversely, other groups exhibit stiffness-driven behavior, where higher load capacity leads to reduced ductility and lower slip.

### Bolt diameter

**Fig. 24:**
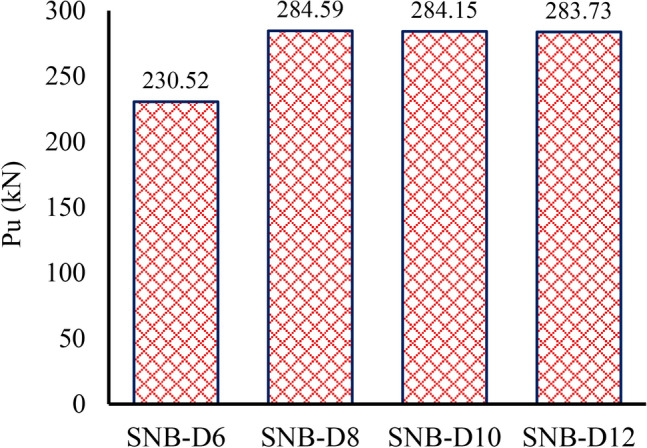
Effect of bolt diameters on the ultimate force per shear connectors.

The bolt diameters of 6 mm, 8 mm, 10 mm, and 12 mm were chosen for the detailed parametric analysis, as shown in Table 7 (group G5). Figure 24 demonstrates how varying bolt diameters influence the ultimate force per shear connector across different specimens. Figure 24 demonstrates that the peak load (${P}_{u}$) for specimens with bolt diameters varying from 6 to 12 mm are 230.42 kN, 284.59 kN, 284.15 kN, and 283.73 kN, respectively. It is evident that for bolt diameters greater than or equal to 8 mm, the peak load value and slip capacity remain constant. Moreover, the predominant failure mode in these specimens is bearing within the TSC section, with no visible damage to the concrete slab or bolted connectors. Given that the TSC thicknesses is 4mm, and to prevent such failures, it is recommended that the bolt diameters shall not exceed two times web thickness of TSC. This aligns with the requirements of the Egyptian code^36^.

The observed trend in Group G5, where increasing the bolt diameter beyond 8 mm does not significantly influence the peak load or slip capacity, can be attributed to the interaction between the bolt, TSC section, and concrete. Initially, as the bolt diameter increases from 6 to 8 mm, the load-bearing capacity improves due to the larger cross-sectional area, which enhances shear resistance. However, for bolt diameters of 8 mm and above, the peak load stabilizes because the failure mode shifts primarily to bearing within the TSC section rather than failure of the bolt or concrete.

This behavior occurs because the TSC thickness (4 mm) becomes a limiting factor. When the bolt diameter exceeds twice the TSC web thickness, localized bearing failure in the TSC section dominates, preventing further improvement in load capacity. Since no significant deformation or damage is observed in the concrete slab or bolted connectors, it confirms that increasing the bolt diameter beyond a certain threshold does not enhance performance but rather leads to premature bearing failure in the TSC section.

To mitigate such failures and optimize load transfer, it is recommended that the bolt diameter should not exceed twice the web thickness of the TSC, aligning with the Egyptian code^36^. This ensures balanced load distribution and prevents excessive localized stress concentrations in the TSC section, which could compromise structural integrity.

## Conclusion

This study investigated the performance of demountable bolted shear connectors in steel–concrete composite structures through experimental and numerical analyses. The findings provide valuable insights into the behavior of these connectors under push-out loading conditions, supported by robust evidence from both experimental tests and finite element simulations. The key outcomes, practical applications, and recommendations are summarized below:

1. Demountable shear studs significantly improved shear capacity, with specimens S_NB_ and S_NBR_ exhibiting a 217% higher peak load than the specimen without studs. This highlights the effectiveness of bolted connectors in enhancing load transfer between steel and concrete.
2. Adding reinforcement to the concrete block (S_NBR_) had minimal impact on peak load but increased peak slip by 37.7% and shear stiffness by 18.7% compared to the unreinforced specimen (S_NB_). This suggests that reinforcement enhances ductility and deformation capacity, contributing to improved overall structural performance.
3. Bolted specimens primarily failed by bolt shank fracture at the steel–concrete interface with localized concrete crushing, while non-bolted specimens (S_N_) exhibited concrete splitting and debonding. This emphasizes the role of shear connectors in preventing premature failure and enhancing structural integrity.
4. Increasing the thickness of the tubular steel column (TSC) significantly improved peak load capacity but reduced slip capacity, indicating a trade-off between strength and ductility.
5. Higher tensile strength of the TSC led to increased peak loads but reduced slip capacity, emphasizing the reduced ductility of high-strength materials.
6. Higher concrete strength marginally increased peak load but reduced slip capacity due to the brittle nature of high-strength concrete.
7. Taller shear studs improved both peak load and slip capacity, enhancing load transfer and deformation capacity.
8. Bolt diameter should not exceed twice the TSC web thickness to prevent bearing failure

### Practical applications

1. Demountable bolted shear connectors enable steel and concrete reuse, making them ideal for temporary structures, modular construction, and future retrofitting.
2. The improved ductility and deformation capacity provided by reinforcement in bolted connectors can enhance the seismic performance of composite structures, making them more resilient to earthquake-induced forces.
3. The enhanced load-bearing capacity and stiffness of bolted connectors make them suitable for high-rise buildings and long-span bridges, where high performance and durability are critical.
4. Demountable connectors can be used in the rehabilitation of existing structures, allowing for the replacement or upgrading of shear connectors without extensive demolition.

### Recommendations for practicing engineers

1. Demountable bolted shear connectors are a viable alternative to welded studs, offering comparable shear strength with the added benefit of reusability. They are particularly suitable for structures requiring disassembly or retrofitting.
2. Engineers should consider incorporating reinforcement to improve the overall performance and resilience of composite structures
3. Use TSC thicknesses greater than half the bolt diameter to prevent bearing failure
4. Avoid excessively high-strength concrete in applications requiring high ductility, as it reduces slip capacity.
5. Optimize bolt diameter to balance load capacity and prevent premature TSC bearing failure.

### Limitations and future research

1. The experimental study was conducted on a limited number of specimens (three) using normal-strength concrete and specific steel grades. Future research should examine demountable connectors with ultra-high-performance concrete (UHPC) and advanced steel alloys to enhance statistical reliability and result reproducibility.
2. The study did not address the long-term durability of bolted connectors under cyclic loading or environmental exposure. Further investigations are needed to evaluate fatigue and corrosion resistance.
3. While the experimental and numerical results are promising, full-scale testing of composite beams or columns with demountable connectors is necessary to validate their performance in real-world applications.
4. A cost–benefit analysis of demountable connectors compared to welded studs, considering lifecycle costs and environmental impact, would provide valuable insights for decision-making.

## Data Availability

The datasets used and/or analysed during the current study available from the corresponding author on reasonable request.

## References

[1] Brozzetti, J. Design development of steel-concrete composite bridges in France. *J. Constr. Steel Res.* **55**, 229–243 (2000).

[2] Nakamura, S.-i, Momiyama, Y., Hosaka, T. & Homma, K. New technologies of steel/concrete composite bridges. *J. Constr. Steel Res.* **58**, 99–130 (2002).

[3] Oehlers, D. J., Bradford, M. A. *Composite Steel and Concrete Structures: Fundamental Behaviour: Composite Steel and Concrete Structures: Fundamental Behaviour* (Elsevier, 2013).

[4] Wu, D., Gao, W., Feng, J. & Luo, K. Structural behaviour evolution of composite steel-concrete curved structure with uncertain creep and shrinkage effects. *Compos. B Eng.* **86**, 261–272 (2016).

[5] Johnson, R. P. *Composite Structures of Steel and Concrete: Beams, Slabs, Columns and Frames for Buildings* (Wiley, 2018).

[6] Abbas, J. L. & Allawi, A. A. Experimental and numerical investigations of composite concrete–steel plate shear walls subjected to axial load. *Civ. Eng. J.* **5**, 2402–2422 (2019).

[7] Laftah Abbas, J. & AbdulMajeed, A. A. Structural behavior of axially loaded composite concrete-steel plate shear walls. *Int. J. Eng.* **32**, 1548–1558 (2019).

[8] Mahmood, E. M., Allawi, A. A. & El-Zohairy, A. Flexural performance of encased pultruded GFRP I-beam with high strength concrete under static loading. *Materials.* **15**, 4519 (2022). 35806644 10.3390/ma15134519 PMC9267526

[9] Shariati, A. Various types of shear connectors in composite structures: A review. *Int. J. Phys. Sci*. **7** (2012).

[10] Zhang, Y. et al. Experimental study on shear behavior of high strength bolt connection in prefabricated steel-concrete composite beam. *Compos. Part B. Eng.* **159**, 481–489 (2019).

[11] Moynihan, M. C. & Allwood, J. M. Viability and performance of demountable composite connectors. *J. Constr. Steel Res.* **99**, 47–56 (2014).

[12] ACI. *ACI 318-19: Building Code Requirements for Structural Concrete *(ACI Farmington Hills, 2019).

[13] En C. 1-1, Eurocode 4: Design of composite steel and concrete structures. Part 1-1: General rules and rules for buildings 2004. (1994).

[14] Ansi B. AISC 360-16, specification for structural steel buildings. Chicago AISC. (2016).

[15] Uy, B., Hicks, S. J., Kang, W.-H., Thai, H.-T., Aslani, F. The new Australia/New Zealand standard on composite steel-concrete buildings, ASNZS2327. In *Proceedings of the Eighth International Conference on Composite Construction in Steel and Concrete: American Institute of Steel Construction*, 86–97 (2017).

[16] Dai, X., Lam, D. & Saveri, E. Effect of concrete strength and stud collar size to shear capacity of demountable shear connectors. *J. Struct. Eng.* **141**, 04015025 (2015).

[17] Pavlović, M., Marković, Z., Veljković, M. & Buđevac, D. Bolted shear connectors vs headed studs behaviour in push-out tests. *J. Constr. Steel Res.* **88**, 134–149 (2013).

[18] Ban, H. et al. Time-dependent behaviour of composite beams with blind bolts under sustained loads. *J. Constr. Steel Res.* **112**, 196–207 (2015).

[19] Pathirana, S. W., Uy, B., Mirza, O. & Zhu, X. Strengthening of existing composite steel-concrete beams utilising bolted shear connectors and welded studs. *J. Constr. Steel Res.* **114**, 417–430 (2015).

[20] Pathirana, S. W., Uy, B., Mirza, O. & Zhu, X. Flexural behaviour of composite steel–concrete beams utilising blind bolt shear connectors. *Eng. Struct.* **114**, 181–194 (2016).

[21] Henderson, I., Zhu, X., Uy, B. & Mirza, O. Dynamic behaviour of steel–concrete composite beams with different types of shear connectors. Part I: Experimental study. *Eng. Struct.* **103**, 298–307 (2015).

[22] Liu, X., Bradford, M. A., Chen, Q.-J. & Ban, H. Finite element modelling of steel–concrete composite beams with high-strength friction-grip bolt shear connectors. *Finite Elem. Anal. Des.* **108**, 54–65 (2016).

[23] Király, K. & Dunai, L. Experimental study of novel demountable shear connectors for steel-concrete composite buildings. *Period. Polytech. Civ. Eng.* **68**, 647–656 (2024).

[24] Hamoda, A., Emara, M. & Mansour, W. Behavior of steel I-beam embedded in normal and steel fiber reinforced concrete incorporating demountable bolted connectors. *Compos. Part B Eng.* **174**, 106996 (2019).

[25] Yang, F., Liu, Y., Jiang, Z. & Xin, H. Shear performance of a novel demountable steel-concrete bolted connector under static push-out tests. *Eng. Struct.* **160**, 133–146 (2018).

[26] Weng, C., Yen, S. & Jiang, M. Experimental study on shear splitting failure of full-scale composite concrete encased steel beams. *J. Struct. Eng.* **128**, 1186–1194 (2002).

[27] Furlong, R. W. Design of steel-encased concrete beam-columns. *J. Struct. Div.* **94**, 267–281 (1968).

[28] El-Tawil, S., Sanz-Picon, C. & Deierlein, G. Evaluation of ACI 318 and AISC (LRFD) strength provisions for composite beam-columns. *J. Constr. Steel Res.* **34**, 103–123 (1995).

[29] Kim, J.-S., Kwark, J., Joh, C., Yoo, S.-W. & Lee, K.-C. Headed stud shear connector for thin ultrahigh-performance concrete bridge deck. *J. Constr. Steel Res.* **108**, 23–30 (2015).

[30] An, L. & Cederwall, K. Push-out tests on studs in high strength and normal strength concrete. *J. Constr. Steel Res.* **36**, 15–29 (1996).

[31] Shim, H., Chung, K., Jang, S., Park, S., Lee, J. Push-out tests on shear studs in high strength concrete. In *Proc 7th International Conference on Fracture Mechanics of Concrete and Concrete Structures (FraMCoS-7), Jeju, Korea*, 06–10 (2010).

[32] Jiang, Y., Hu, X., Hong, W. & Wang, B. Experimental study and theoretical analysis of partially encased continuous composite beams. *J. Constr. Steel Res.* **117**, 152–160 (2016).

[33] Shim, C.-S., Lee, P.-G. & Yoon, T.-Y. Static behavior of large stud shear connectors. *Eng. Struct.* **26**, 1853–1860 (2004).

[34] Lin, Z., Liu, Y. & He, J. Behavior of stud connectors under combined shear and tension loads. *Eng. Struct.* **81**, 362–376 (2014).

[35] Kozma, A., Odenbreit, C., Braun, M. V., Veljkovic, M. & Nijgh, M. *Push-Out Tests on Demountable Shear Connectors of Steel-Concrete Composite Structures* 45–54 (Elsevier, 2019).

[36] Egyptian code of practice for steel construction and bridges (allowable stress design-ASD) code No. (205) Ministerial Decree No 279-2001 Permanent Committee for the code of practice for steel construction and bridges.

[37] Fayed, S. et al. Shear strengthening of RC beams using prestressed near-surface mounted bars reducing the probability of construction failure risk. *Materials.* **17**, 5701 (2024). 39685136 10.3390/ma17235701 PMC11642131

[38] Yehia, S. A., Shahin, R. I. & Fayed, S. Compressive behavior of eco-friendly concrete containing glass waste and recycled concrete aggregate using experimental investigation and machine learning techniques. *Constr. Build. Mater.* **436**, 137002 (2024).

[39] ASTM Standard B928/B928M, Standard Specification for High Magnesium Aluminum-Alloy Products for Marine Service and Similar Environments, ASTM International, West Conshohocken, PA, 2002.

[40] Xu, C., Sugiura, K., Wu, C. & Su, Q. Parametrical static analysis on group studs with typical push-out tests. *J. Constr. Steel Res.* **72**, 84–96 (2012).

[41] Hamoda, A. et al. Experimental and numerical investigations of the shear performance of reinforced concrete deep beams strengthened with hybrid SHCC-mesh. *Case Stud. Constr. Mater.* **21**, e03495 (2024).

[42] Hamoda, A., Shahin, R., Ahmed, M., Abadel, A., Yehia, S. Flexural behavior of normal concrete circular beams strengthened using engineered cementitious composite and stainless steel tube. *Mag. Concr. Res*. 1–57 (2024).

[43] Hamoda, A. et al. *Strengthening of Reinforced Concrete Columns Incorporating Different Configurations of Stainless-Steel Plates* 106577 (Elsevier, 2024).

[44] Hamoda, A., Shahin, R. I., Ahmed, M., Abadel, A. A., Yehia, S. A. Flexural behaviour of normal concrete circular beams strengthened with ECC and stainless steel tubes. *Mag. Concr. Res*. 1–18 (2024).

[45] Hamoda, A. et al. Experimental and numerical analysis of deep beams with openings strengthened with galvanized corrugated and flat steel sheets. *Case Stud. Constr. Mater.* **21**, e03522 (2024).

[46] Sennah, K., Hamoda, A., Abadel, A., Yehia, S., Shahin, R. Shear strengthening of simply-supported deep beams with openings incorporating combined steel-reinforced engineered cementitious composites and externally bonded carbon fibre-reinforced polymer sheets. *Mag. Concr. Res*. 1–42 (2024).

[47] dos Santos, L. R., de Sousa, C. H., Caldas, R. B. & Grilo, L. F. Finite element model for bolted shear connectors in concrete-filled steel tubular columns. *Eng. Struct.* **203**, 109863 (2020).

[48] He, J., Suwaed, A. S. & Vasdravellis, G. *Horizontal Pushout Tests and Parametric Analyses of a Locking-Bolt Demountable Shear Connector* 667–683 (Elsevier, 2022).

[49] Fang, Z. et al. Shear performance of high-strength friction-grip bolted shear connector in prefabricated steel–UHPC composite beams: Finite element modelling and parametric study. *Case Stud. Constr. Mater.* **18**, e01860 (2023).

[50] Mander, J. B., Priestley, M. J. & Park, R. Theoretical stress-strain model for confined concrete. *J. Struct. Eng.* **114**, 1804–1826 (1988).

[51] Yong, Y.-K., Nour, M. G. & Nawy, E. G. Behavior of laterally confined high-strength concrete under axial loads. *J. Struct. Eng.* **114**, 332–351 (1988).

[52] Saenz, L. Discussion of Paper By Desai P, Krishnan S. Equation for stress-strain curve of concrete. *J. ACI. Proc.* 61 (1964).

[53] ACI318-11. *Building Code Requirements for Structural Concrete and Commentary (ACI 318-11).* (American Concrete Institute, 2011).

[54] Hu, H.-T. & Schnobrich, W. C. Constitutive modeling of concrete by using nonassociated plasticity. *J. Mater. Civ. Eng.* **1**, 199–216 (1989).

[55] Nayal, R. & Rasheed, H. A. Tension stiffening model for concrete beams reinforced with steel and FRP bars. *J. Mater. Civ. Eng.* **18**, 831–841 (2006).

[56] Wahalathantri, B., Thambiratnam, D., Chan, T., Fawzia, S. A material model for flexural crack simulation in reinforced concrete elements using ABAQUS. In *Proceedings of the First International Conference on Engineering, Designing and Developing the Built Environment for Sustainable Wellbeing: Queensland University of Technology*, 260–264 (2011).

[57] ABAQUS User Manual. Version 6.9. (DS SIMULIA Corp, 2009).

[58] Jankowiak, T. & Lodygowski, T. Identification of parameters of concrete damage plasticity constitutive model. *Found. Civ. Environ. Eng.* **6**, 53–69 (2005).

[59] Yehia, S. A., Fayed, S., Shahin, R. I. & Ahmed, R. B. Effect of existing holes under the loading plate on local compressive strength of plain concrete blocks: An experimental and numerical study. *Case Stud. Constr. Mater.* **21**, e03937 (2024).

[60] Shahin, R. I., Ahmed, M., Liang, Q. Q. & Yehia, S. A. Predicting the web crippling capacity of cold-formed steel lipped channels using hybrid machine learning techniques. *Eng. Struct.* **309**, 118061 (2024).

[61] ABAQUS Analysis User’S Manual-Version 6.14-2, ABAQUS Inc., USA, 2018.

[62] Trattnig, G., Antretter, T. & Pippan, R. Fracture of austenitic steel subject to a wide range of stress triaxiality ratios and crack deformation modes. *Eng. Fract. Mech.* **75**, 223–235 (2008).

[63] Rice, J. R. & Tracey, D. M. On the ductile enlargement of voids in triaxial stress fields∗. *J. Mech. Phys. Solids.* **17**, 201–217 (1969).

[64] Lemaitre, J. *A Continuous Damage Mechanics Model for Ductile Fracture.* (1985).

[65] Bonora, N., Ruggiero, A., Esposito, L. & Gentile, D. CDM modeling of ductile failure in ferritic steels: assessment of the geometry transferability of model parameters. *Int. J. Plast.* **22**, 2015–2047 (2006).

[66] Hooputra, H., Gese, H., Dell, H. & Werner, H. A comprehensive failure model for crashworthiness simulation of aluminium extrusions. *Int. J. Crashworthiness.* **9**, 449–464 (2004).

[67] EN1993-1-3, Eurocode 3: Design of Steel Structures: Part 1–3: Gerneral Rules: Supplementary Rules for Cold-Formed Members and Sheeting, European Committee for Standardization, Brussels, Belgium, 2006.

[68] Yun, X. & Gardner, L. Stress–strain curves for hot-rolled steels. *J. Constr. Steel Res.* **133**, 36–46 (2017).

